# Detection of microRNA Expression in Human Peripheral Blood Microvesicles

**DOI:** 10.1371/journal.pone.0003694

**Published:** 2008-11-11

**Authors:** Melissa Piper Hunter, Noura Ismail, Xiaoli Zhang, Baltazar D. Aguda, Eun Joo Lee, Lianbo Yu, Tao Xiao, Jeffrey Schafer, Mei-Ling Ting Lee, Thomas D. Schmittgen, S. Patrick Nana-Sinkam, David Jarjoura, Clay B. Marsh

**Affiliations:** 1 Division of Pulmonary, Allergy, Critical Care, Sleep Medicine, College of Medicine, The Ohio State University, Columbus, Ohio, United States of America; 2 Molecular, Cellular, Developmental Biology Program, College of Biological Sciences, The Ohio State University, Columbus, Ohio, United States of America; 3 Center for Biostatistics, College of Medicine, The Ohio State University, Columbus, Ohio, United States of America; 4 Mathematical Biosciences Institute, College of Medicine, The Ohio State University, Columbus, Ohio, United States of America; 5 College of Pharmacy, The Ohio State University, Columbus, Ohio, United States of America; 6 Biostatistics, College of Public Health, The Ohio State University, Columbus, Ohio, United States of America; 7 Department of Epidemiology and Biostatistics, University of Maryland, College Park, Maryland, United States of America; Chinese University of Hong Kong, Hong Kong

## Abstract

**Background:**

MicroRNAs (miRNA) are small non-coding RNAs that regulate translation of mRNA and protein. Loss or enhanced expression of miRNAs is associated with several diseases, including cancer. However, the identification of circulating miRNA in healthy donors is not well characterized. Microvesicles, also known as exosomes or microparticles, circulate in the peripheral blood and can stimulate cellular signaling. In this study, we hypothesized that under normal healthy conditions, microvesicles contain miRNAs, contributing to biological homeostasis.

**Methodology/Principal Findings:**

Microvesicles were isolated from the plasma of normal healthy individuals. RNA was isolated from both the microvesicles and matched mononuclear cells and profiled for 420 known mature miRNAs by real-time PCR. Hierarchical clustering of the data sets indicated significant differences in miRNA expression between peripheral blood mononuclear cells (PBMC) and plasma microvesicles. We observed 71 miRNAs co-expressed between microvesicles and PBMC. Notably, we found 33 and 4 significantly differentially expressed miRNAs in the plasma microvesicles and mononuclear cells, respectively. Prediction of the gene targets and associated biological pathways regulated by the detected miRNAs was performed. The majority of the miRNAs expressed in the microvesicles from the blood were predicted to regulate cellular differentiation of blood cells and metabolic pathways. Interestingly, a select few miRNAs were also predicted to be important modulators of immune function.

**Conclusions:**

This study is the first to identify and define miRNA expression in circulating plasma microvesicles of normal subjects. The data generated from this study provides a basis for future studies to determine the predictive role of peripheral blood miRNA signatures in human disease and will enable the definition of the biological processes regulated by these miRNA.

## Introduction

MicroRNAs (miRNAs) are small non-coding RNA sequences of about 22 nt [1]. miRNAs have been identified in animals, plants and viruses [2]. To date, advances in methodology have led to the identification of 587 human miRNAs (Sanger updated database [3]), and it is estimated that over 30% of human genes are targeted by these miRNAs [4]. miRNAs are implicated in fundamental cellular processes including hematopoietic differentiation [5], [6], cell cycle regulation [7], [8] and metabolism [9], [10]. The evolutionary conserved miRNAs are transcribed by polymerase II and function as post-transcriptional gene regulators by targeting messenger RNA (mRNA) or proteins [11]. All precursor miRNA have stem loop structures, cleaved by Drosha and Dicer, to form the mature functional miRNA. Through formation of RNA-induced silencing complexes, miRNAs can either cleave mature mRNA molecules or inhibit their translation thus representing a newly discovered layer of gene regulation.

MiRNAs play an important role in disease progression and carcinogenesis [12], [13]; MiRNAs can act as oncogenes or tumor suppressors [14]. A study led by Calin *et al.* first revealed the connection between miR-15 and -16 and B-cell chronic lymphocytic leukemia [15]. Later, several studies showed the importance of miRNA in prognosis, initiation and progression of numerous cancer types [16]–[18]. Hence, utilizing miRNA fingerprinting as a novel diagnostic, prognostic and disease surveillance tool is of increasing importance [19].

Recent evidence reveals that genetic exchange of mRNA and miRNA between cells can be accomplished through microvesicles, or exosome-mediated transfer [20]. Microvesicles are small exosomes/vesicles of endocytic origin released by normal healthy or damaged cell types [21]–[23]. Alternative names for microvesicles provide insight into their source. Microparticles are microvesicles released from activated platelets; exosomes are microvesicles secreted by tumors; and ectosomes are neutrophil-derived microvesicles [23]. Current estimates of microvesicle concentration in the peripheral blood of healthy individuals are 5–50 µg/ml. Microvesicles are shed from the plasma membrane into the extracellular environment to facilitate communication between cells. Despite their small size (50 nm to 1 µm) microvesicles are enriched in bioactive molecules and contain nucleic acid and/or protein; these cell particles play a role in growth, differentiation and cancer progression [24]. Tumor-derived microvesicles transfer mRNA to monocytes within the tumor microenvironment to activate these cells to produce cytokines to enhance tumor growth and dampen the immune response [25]. Deregibus et al report that mRNA from endothelial-derived MV are pro-angiogenic [26]. In the peripheral blood, two-thirds of microvesicles are derived from platelets. Platelet-derived microvesicles play a role in angiogenesis and the metastatic spread of cancers such as lung cancer [27]. Platelet-derived microvesicles induce an immune response upon regulating gene expression in hematopoietic, endothelial, and monocytic cells [28], [29]. Notably, platelet-derived microvesicle subpopulations are increased in patients with sepsis [30], [31], whereas patients with pulmonary arterial hypertension have increased endothelial-derived microvesicles [32]. However it is currently unknown whether microvesicle content changes in theses diseases.

Interestingly, a connection between microvesicles and miRNA has been recently made. Recently, Valadi and colleagues reported that vesicles released from human and murine mast cell lines contain over 1200 mRNA and approximately 121 miRNA molecules [20].

Considerable evidence demonstrates the importance of miRNA as an inevitable cornerstone of the human genetic system. Employing the use of microvesicles to transfer genetic material would be an efficient transfer method within the human body. Microvesicles containing miRNAs would enable intercellular and inter-organ communication in the body. Here, we explored the hypothesis that miRNAs are contained within microvesicles in the peripheral blood. We examined the expression of miRNAs from the peripheral blood microvesicles and PBMC from normal human volunteers. Our study identifies miRNAs in circulating microvesicles in the plasma and in PBMC and characterizes specific miRNA expression.

## Methods

### Blood collection and microvesicle isolation

Peripheral blood (40 cc) was collected in EDTA tubes from 24 female and 27 male healthy non-smoking Caucasian donors following informed consent. Written consent was obtained from all subjects in accordance with approved Institution Review Board protocol (1978H0059) and HIPAA regulations. Criteria for volunteers consist of no recent illness or treatment for a chronic medical condition. No medical history was obtained from donors. Collection of the blood occurred between morning and early afternoon. The average age for female and male donors was 35±12 and 31±9, respectively, while the median age for both female and male donors was 29. The peripheral blood was diluted 1∶1 with sterile low endotoxin PBS (Mediatech, Inc. Manassas, VA), layered over ficoll-hypaque (d = 1.077) (Mediatech, Inc.), and centrifuged as previously described [33]. The mononuclear cell fraction was washed once in PBS. The microvesicles were purified from the plasma. Briefly, the vesicles were concentrated by centrifugation at 160,000× g for 1 hr at 4°C [34]. To isolate platelets, blood was collected in 3.8% sodium citrate tubes from six donors, using a washing method as previously described [35], [36]. Following centrifugation of the blood, the platelet rich plasma was incubated with 1 µM of PGE_1_ (Sigma-Aldrich, St. Louis, MO). The platelets were washed twice in Tyrodes buffer containing 138 mM NaCl, 2.9 mM KCl, 12 mM NaHCO_3_, 0.36 mM NaHPO_4_, 5.5 mM glucose, 1.8 mM CaCl_2_, and 0.49 mM MgCl_2_, pH 6.5. The platelets were then washed one additional time in Tyrodes buffer, pH 7.4.

### RNA Extraction

Total RNA was isolated by Trizol (Invitrogen, Carlsbad, CA) extraction method. To increase the yield of small RNAs, the RNA was precipitated overnight. RNA concentration and RNA integrity were determined by capillary electrophoresis on an Agilent 2100 Bioanalyzer (Agilent Technologies, Inc, Santa Clara, CA). We recovered an average 1.740 µg of total RNA from the plasma fractions. For RNA isolated from mononuclear cells, only a RNA integrity number (RIN)≥9 was used along with its matched plasma sample for profiling. Since the intact 18s and 28s rRNA were variable in the microvesicles, the RIN was not a constraint for these samples, however we observed a RIN between 2–8.

### miRNA profiling by quantitative PCR

At the time of the study, commercially available primers were available for 420 mature human miRNAs. We used these looped primers to profile 420 mature miRNAs by real-time PCR using an Applied Biosystems 7900HT real-time PCR instrument equipped with a 384 well reaction plate. RNA (500 ng) was converted to cDNA by priming with a mixture of looped primers (Mega Plex kit, Applied Biosystems, Foster City, CA) using previously published reverse transcription conditions [37]. As there is no known control miRNA in microvesicles, several internal controls were examined. Primers to the internal controls, small nucleolar (sno)RNA U38B, snoRNA U43, small nuclear (sn)RNA U6 as well as 18S and 5S rRNA were included in the mix of primers. Liquid-handling robots and the Zymak Twister robot were used to increase throughput and reduce error. Real-time PCR was performed using standard conditions.

### Flow Cytometry

Peripheral blood microvesicles were directly immunostained from plasma without concentration by centrifugation. To determine the cellular origin, 0.5 cc plasma was immunostained per panel of antibodies. Panel I contained antibodies recognizing CD66b-FITC (neutrophil) (BD Biosciences, San Jose, CA), CD202b (Tie2)-PE (endothelial) (R&D Systems, Minneapolis, MN), CD206 PE-Cy5 (macrophage/dendritic) (BD Biosciences), CD79a-APC (B-cell) (BD Biosciences), and CD14 Pe-Cy7 (monocyte) (BD Biosciences). Panel II contained antibodies to CD41a-PE-Cy5 (platelet) (BD Biosciences), CCR2-APC (monocyte) (R&D Systems), CCR3-PE (dendritic cell) (Caltag, Carlsbad, CA), CCR5-PE-Cy7 (macrophage) (BD Biosciences), and CD3-Alexa 610 (T-cell) (BD Biosciences). Panel III contained isotype control antibodies (BD Biosciences). All antibodies were used according to manufacturers' recommendation. To confirm that the microvesicles were the correct size, flow cytometry gates were set using 2 micron bead standards (BD Biosciences). The samples were analyzed on BD Aria flow cytometer (BD Biosciences). Data were expressed as percent of gated events.

### Statistical analysis

Since C_T_ scores greater than 35 are considered non-specific [37], miRNAs in which 80% of individual observations had a raw C_T_ score greater than 35 were not included in the final data analysis. Using these filtering criteria, we removed 128 and 152 miRNAs for the plasma and PBMC samples, respectively, from analysis. The internal controls (18S, 5S, snoRNA U38B, snoRNA U43, and snRNA U6) were highly variable in the plasma microvesicles and were significantly different in plasma microvesicles versus peripheral blood mononuclear cells (PBMC). To reduce bias caused by using an arbitrary miRNA as a normalization correction factor, the miRNAs were compared between plasma microvesicles and PBMC based on their relative expression to the overall miRNA expression on each array using median normalization analysis [33]. To test the difference of specific miRNA between plasma microvesicles and PBMC, linear mixed models were used and p-values were generated from the model based on the estimated difference and sample variation. Fold-change was calculated based on the estimated mean difference (2^∧(−ΔCT)^). MiRNAs were subjected to hierarchical clustering using Euclidean distance based on their relative mean expression. MiRNAs were also ranked based on their raw C_T_ score for each plasma microvesicles and PBMC.

### Pathway analysis and prediction

Predicted miRNA targets were determined using the miRanda algorithm (http://microrna.sanger.ac.uk/targets/v5/) and TargetScan v4.2 (http://www.targetscan.org/). Common predicted targets as well as targets from each database were subjected to pathway exploration using the Ingenuity Pathway Analysis (IPA) software (Ingenuity Systems, Redwood City, CA). To avoid exceeding the maximum gene list size allowed by IPA program, we limited targets based on each programs assigned score. Therefore scores of at least 17 and −0.31 were considered for miRanda and TargetScan, respectively. Using this software and its accompanying interaction database, top-ranked pathways were determined based on the incidence of predicted miRNA targets in a list of canonical pathways provided by the software. IPA also generates the top-ranked networks where the predicted miRNA targets are found; according to gene ontology, the biological functions associated with these networks are provided.

## Results

### Peripheral blood microvesicle subpopulations

**Figure 1:**
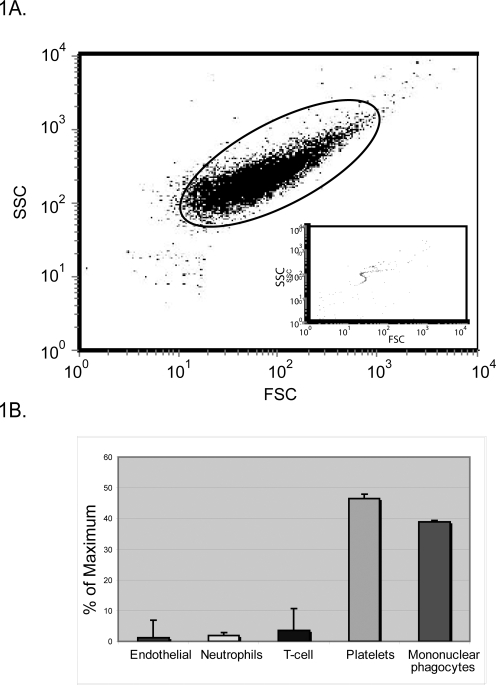
Analysis of the origin of peripheral blood microvesicles. Peripheral blood microvesicles from healthy donors (n = 10) were analyzed by flow cytometry. (A) The size of the microvesicles was determined by FSC vs. SSC. The gate was set according to 2 micron beads (inset). (B) To determine cell origin, microvesicles were stained for CD3, CD202b (Tie-2), CD66b, CD79a, or CD41a to determine those that originated from T-cells, endothelial cells, neutrophils, B-cells, or platelets. Mononuclear phagocyte-derived microvesicles were positive for CD14, CD206, CCR3, CCR2, or CCR5. Shown is the average % maximum of total gated events±S.E.M.

To ensure that our isolation method purified microvesicles, we examined size of the microvesicles by flow cytomtery. To visualize the microvesicles by flow cytometry, the axis for forward scatter (FSC) vs. side scatter (SSC) plot required log-scale (Figure 1A). Flow cytometry gates were set using 2 micron beads (Figure 1A, inset). Notably, the size of the microvesicles analyzed in this study was in the range of the bead standards. We also examined the cellular origin of microvesicles within the peripheral blood of normal healthy individuals. Using flow cytometry, we found that the majority of the peripheral blood microvesicles are platelet-derived (Figure 1B), as previously reported [34]. We also observed a second large population of microvesicles that were derived from mononuclear phagocyte cell lineage. This population was immunostained with antibodies that detected surface antigens on mononuclear phagocytes. Notably, only a small percentage of the peripheral blood microvesicles were derived from T-cells and neutrophils. We failed to detect vesicles that originated from B-cells (data not shown). Of interest, we detected a small subpopulation of microvesicles that expressed surface antigens from endothelial cells.

### miRNA expression in plasma microvesicles and PBMC

**Figure 2:**
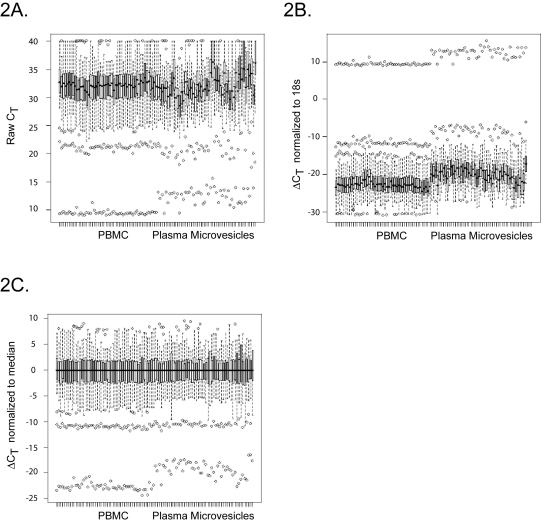
Distribution of miRNA expression. The box plots were generated to demonstrate the distribution of miRNA expression from each PBMC and plasma microvesicle sample. The bottom and top of the boxes are the 25^th^ and 75^th^ percentile (the lower and upper quartiles, respectively), and the band near the middle of the boxes are the 50^th^ percentile (the median). The ends of the whiskers represent the minimum and maximum of the data. (A) Raw C_T_ score of the data from individual donor were plotted. (B). 18s standardized data (ΔC_T_ ) were plotted: ΔC_T_ = raw C_T_ of PBMC or plasma microvesicles – C_T_ of 18S. (C). Median normalized data of each donor was used.

**Figure 3:**
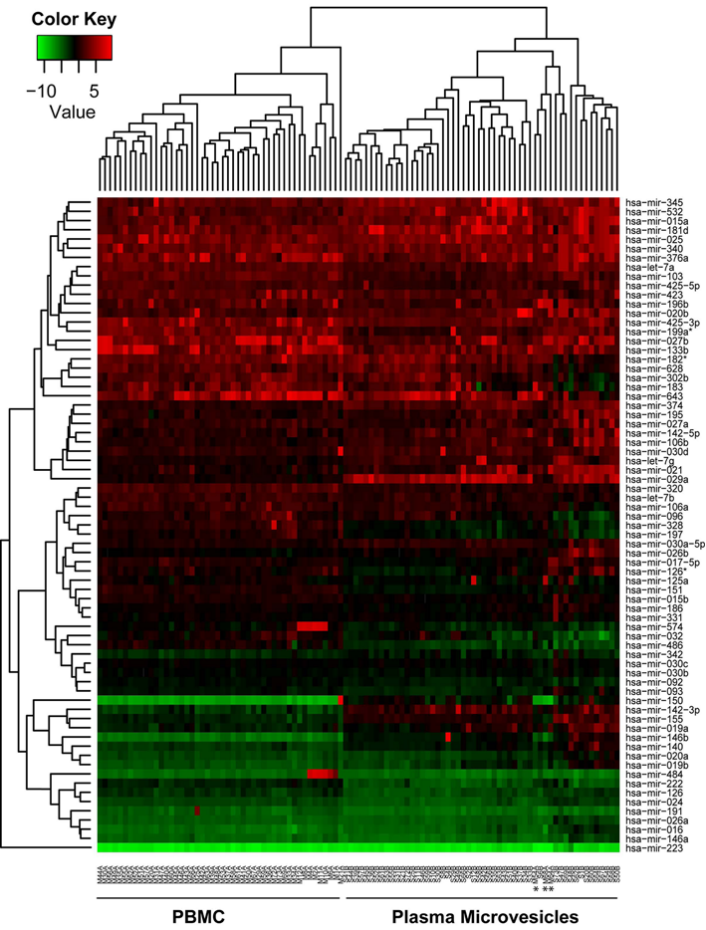
miRNA expression from peripheral blood microvesicles and PBMC. Hierarchical cluster analysis for microvesicles and PBMC is shown based on filtering criteria. The (*) denotes the three PBMC samples which clustered with the plasma microvesicle samples.

**Figure 4:**
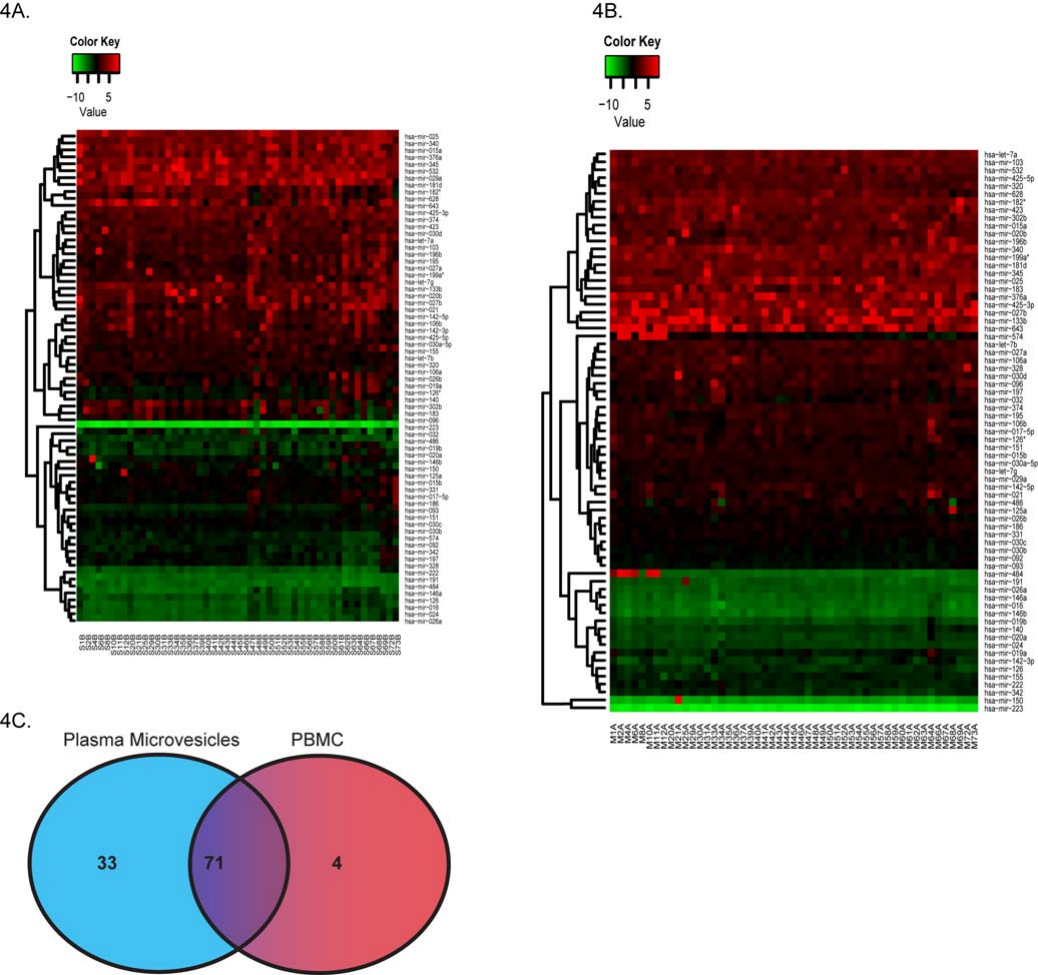
Expression profile of common miRNAs. Heat-maps demonstrating the expression profile for microvesicles (A) and PBMC (B) were generated for miRNAs that were commonly expressed. (C) The number of shared and specific miRNAs for each sample group is shown.

To test our hypothesis that miRNAs are contained in the microvesicle compartment within the peripheral blood to enable communication and influence genetic changes between different tissues within the body, we performed miRNA profiling on the purified microvesicles from all cellular origins in the plasma. We analyzed all subpopulations of microvesicles from 51 non-smoking healthy individuals comprising of 27 males and 24 females. Since we hypothesized that there would be differences in miRNA expression between microvesicles and PBMC, we also purified the PBMC from each donor. Real-time PCR analysis was performed to examine the expression of 420 miRNAs. Expression of the internal controls (18S, 5S, snoRNA U38B, snoRNA U43, and snRNA U6) was highly variable in the plasma microvesicles versus peripheral blood mononuclear cells (PBMC) (Figure 2A and 2B). To reduce bias caused by using an arbitrary miRNA as a normalization correction factor and to reduce the sample variations among qRT-PCR arrays, the miRNAs were compared between plasma microvesicles and PBMC based on their relative expression to the overall miRNA expression on each array using median normalization analysis (Figure 2C) [33]. The filtered and normalized data were subjected to hierarchical cluster analysis comparing the miRNA expression profile between the PBMC and plasma microvesicles samples (Figure 3). All but three PBMC samples clustered separately from the microvesicle samples indicating that the miRNA expression profile between the two groups was significantly different. It is not readily apparent why three samples clustered differently. These donors varied in age and gender. However, based on filtering criteria, we found 104 and 75 miRNAs were significantly expressed in microvesicle and PBMC samples, respectively (Figures 4A and 4B). Of these miRNAs, 71 were co-expressed among each sample group (Figure 4C).

### Age and gender effects

We did not observe age and/or gender effects in miRNA expression from either sample group or across PBMC and plasma microvesicles samples. Notably, the median age for both female and male donors was 29 years. The oldest individual was 58 years old, while the youngest was 21 years of age. Thus, we further stratified the data to examine differences. For each PBMC and microvesicles compartment, we also compared the miRNA expression of the donors with the upper quartile of age to those with the lower quartile of age (mean age for each group was 48.9±6.2 and 21.9±1.2, respectively). However, we failed to detect significant differences in miRNA expression between the samples sets based on age (data not shown).

### Comparison of miRNA expression in PBMC and microvesicles

In Table 1, miRNA expression was analyzed separately in plasma microvesicles and PBMC to determine the most highly expressed miRNAs and to determine the frequency of expression across all donors. Normalized expression for all miRNAs in the plasma microvesicles and PBMC from all tested individuals can be found in Table S1. miR-223, -484, -191, -146a, -16, -26a, -222, -24, -126, and -32 were detected in plasma microvesicles, while miR-150, -146b, -19b and -20a were among the highest miRNAs detected in PBMC, but not in plasma microvesicles. In contrast, the PBMC samples, shared 6 common miRNAs with plasma microvesicles (miR-223, -484, -191, -146a, -16, and -26a). PBMCs also lacked high expression of miR-222, -24, -126, and -32 in the top expressed miRNAs compared to those found in plasma microvesicles.

**Table 1:** Normalized expression and rank analysis of miRNAs in plasma microvesicles or PBMC.

Plasma Microvesicles				PBMC			
miRNA	Normalized Expression (±S.D.)	Frequency Detected Among Donors (%)	Top Ten Ranking (%)	miRNA	Normalized Expression (±S.D.)	Frequency Detected Among Donors (%)	Top Ten Ranking (%)
**miR-223**	1589±653	100	100	**miR-223**	2143±499	100	100
**miR-484**	50.9±22.9	100	96	**miR-150**	241±94.6	98	98
**miR-191**	46.4±14.9	100	100	**miR-146b**	57.5±21.1	100	100
**miR-146**a	39.5±19	100	88	**miR-016**	54.7±32.9	100	100
**miR-016**	25.4±13.3	100	78	**miR-484**	40.6±18.8	89	88
**miR-026**a	25.2±9.95	100	90	**miR-146a**	39.6±13.0	100	98
**miR-222**	24.5±12.4	100	76	**miR-191**	32.4±15.6	100	94
**miR-024**	22.7±10.5	100	80	**miR-026a**	30±8.92	100	100
**miR-126**	18.2±8.04	100	66	**miR-019b**	21.7±7.49	100	80
**miR-032**	15.3±32.6	100	31	**miR-020a**	15±5.11	100	4

Expression of miRNAs in the plasma microvesicles and PBMC were analyzed separately (n = 51). The normalized miRNA expression was generated using median normalization analysis then the mean C_T_ value was converted to expression (2^∧(−ΔCT)^) ±standard deviation (S.D.). Shown is the “Frequency Detected Among Donors” represents the percentage of donors in which the miRNA was detectable by qRT-PCR. The “Top Ten Ranking” indicates the percentage of individuals in which the normalized expression for each miRNA fell within the top ten highly expressed miRNAs.

Based on ranking analysis, miR-223 was the most highly expressed miRNA in both PBMC and plasma microvesicles. We next hypothesized that miRNAs originating from specific organ sites would be detected in the PBMC or plasma microvesicles. Lee et al reported that miR-122a is specifically expressed in liver tissue while miR-216 and miR-217 are preferentially expressed in the pancreas [38]. Interestingly these miRNAs were not identified in the plasma microvesicles (Table S1).

We next examined the predicted function of the most highly expressed miRNAs in the plasma microvesicles and PBMC. We examined the top nine expressed miRNAs from the plasma microvesicles and PBMC samples. Using the Sanger miRBase Target v5 and TargetScan v4.2, we found 1578 and 1899 predicted targets, respectively, for the group of highly expressed miRNAs from plasma microvesicles (Table S2). Merging targets from the two databases, we found 94 common predicted targets. These combined targets were subjected to computational analysis to predict pathways that they would collectively regulate. Using Ingenuity Pathway Analysis (IPA) software, we found canonical pathways involved in metabolism, estrogen receptor signaling, and degradation of phospholipids that were predicted to be highly regulated by these miRNAs (Table 2). Of the nine miRNAs examined from the PBMC fraction, we found 1857 and 2072 predicted mRNA targets from Sanger miRBase and TargetScan database, respectively (Table S2) and 89 common targets (Table S2). The top five canonical pathways expected to be regulated by these common predicted miRNA targets are amino acid and lipid metabolic pathways, among other**s** (Table 2).

**Table 2:** Predicted pathways regulated by miRNAs expressed in the plasma microvesicles and PBMC fractions.

Plasma Microvesicles	p-value	PBMC	p-value
glycerophospholipid metabolism	3.29E-03	axonal guidance signaling	1.47E-02
inositol phosphate metabolism	5.77E-03	synaptic long-term potentiation	2.07E-02
phospholipid degradation	9.17E-03	estrogen receptor signaling	2.23E-02
alanine and aspartate metabolism	1.96E-02	glycerophospholipid metabolism	2.45E-02
estrogen receptor signaling	2.14E-02	D-glutamine and D-glutamate metabolism	2.78E-02

Predicted targets were compiled separately using Sanger miRBase and TargetScan for the top nine miRNAs expressed in the plasma microvesicles and PBMC (Table 1). The common predicted targets were subsequently analyzed by IPA software to determine predicted pathways regulated by the miRNAs. Fisher's exact test was used to calculate a p-value determining the probability of the association between the genes in the dataset and the canonical pathway.

We next examined miRNAs that were differentially expressed between plasma microvesicles and PBMC. We found 22 miRNAs had more than a two-fold increase in expression in the PBMC fraction compared to the plasma microvesicles samples, and among them, 15 miRNAs had more than 3-fold changes compared to PBMC (Table 3, Table S3). Interestingly, none of these differentially expressed miRNAs in plasma microvesicles were identified as tissue-specific miRNAs. As shown in Table 3, miR-486 is the highest differentially expressed miRNA in the plasma microvesicles compared to PBMC. The canonical pathways predicted to be regulated by miR-486 are phenylalanine and cyanoamino acid metabolism, insulin receptor signaling, antigen presentation, and pentose phosphate pathways.

**Table 3:** Comparison of miRNA expression between plasma microvesicles and PBMC.

Expression in Plasma Microvesicles	Fold Change	p-Value	Expression in PBMC	Fold Change	p-Value
**miR-486**	6.742	4.31E-20	**miR-150**	59.93	7.00E-21
**miR-328**	6.602	1.64E-21	**miR-029a**	40.31	9.69E-28
**miR-183**	5.979	7.17E-08	**miR-142-3p**	23.49	1.90E-22
**miR-032**	4.530	8.52E-09	**miR-146b**	22.85	1.81E-22
**miR-574**	4.390	3.05E-06	**miR-155**	9.252	6.61E-23
**miR-027b**	4.208	1.20E-05	**miR-532**	4.730	6.14E-10
**miR-222**	3.995	1.18E-21	**miR-019a**	4.682	2.85E-12
**miR-197**	3.783	5.34E-18	**miR-140**	4.219	2.87E-17
**miR-151**	3.482	2.24E-21	**miR-021**	4.211	4.23E-08
miR-199a*	3.454	3.21E-09	**miR-374**	3.769	8.00E-15
**miR-133b**	3.348	4.05E-06	**miR-181d**	3.713	5.30E-07
**miR-320**	2.637	1.63E-16	**miR-345**	3.670	1.25E-08
**miR-096**	2.270	3.86E-4	**let-7g**	3.247	4.99E-09
**miR-103**	2.190	1.99E-08	**miR-015a**	3.226	3.24E-07
**miR-017-5p**	2.037	1.26E-07	**miR-019b**	3.213	7.11E-12
			**miR-142-5p**	2.671	9.83E-08
			**miR-106b**	2.491	1.28E-07
			**miR-016**	2.392	1.35E-10
			**miR-026b**	2.307	5.44E-06
			**miR-195**	2.243	5.50E-5

In order to test the differences of miRNA expression between plasma microvesicles and PBMC, data were analyzed using linear mixed models. The p-values were generated from the model based on the estimated difference and sample variation. A bonferroni adjustment for multiple comparisons (72 comparisons) to control type I error to reduce the number of false positives was performed, therefore “p-value” was considered as significant if p-value<0.05/72 = 0.0006. Fold-change was calculated based on the estimated mean difference (2^∧(−ΔCT)^). Shown are miRNAs that were significantly expressed at greater than 2-fold.

### Comparison of miRNA expression in platelet and plasma microvesicles

**Figure 5:**
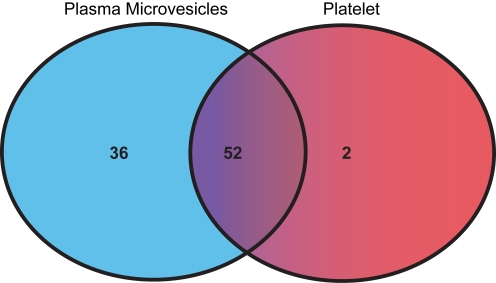
Venn diagram comparing miRNA expression in platelets and plasma microvesicles. The number of shared and specific miRNAs for each sample group is shown.

Since the majority of plasma microvesicles were platelet-derived, we also isolated platelets from several of the donors (n = 6) to profile miRNA expression. We found 52 co-expressed miRNAs in plasma microvesicles and platelets, while there were many more miRNAs unique to the plasma microvesicles compared to platelets (Figure 5). As shown in Table 4, there are many similarities between the platelets and the plasma microvesicles and PBMC. One striking observations is that miR-223 is also the highest expressed miRNA in platelets. Of interest, we failed to observe the expression of miR-484 in the platelets, which is found in both plasma microvesicles and PBMC.

**Table 4:** Normalized miRNA expression in platelets.

detector_name	Average Normalized Expression	Standard Deviation
**miR-484**	21.079	5.936
**miR-191**	19.282	2.716
**miR-146a**	14.438	4.915
**miR-016**	12.851	3.097
**miR-026a**	11.325	2.159
**miR-126**	10.944	4.664
**miR-024**	8.910	2.883
**miR-020a**	7.392	1.640
**miR-019b**	7.287	2.611
**miR-222**	6.626	2.071
**miR-032**	5.551	5.855
**miR-486**	4.510	3.876
**miR-643**	4.391	4.507
**miR-150**	2.408	2.029
**miR-096**	2.113	3.044
**miR-146b**	1.936	0.538
**miR-574**	1.832	0.631
**miR-140**	1.695	0.268
**miR-151**	1.607	0.670
**miR-093**	1.543	0.738
**miR-183**	1.421	2.232
**miR-092**	1.418	0.581
**miR-142-3p**	1.364	0.602
**miR-328**	1.298	0.324
**miR-019a**	1.195	0.514
**miR-030c**	1.124	0.411
**miR-186**	1.096	0.346
**miR-197**	1.092	0.447
**miR-030b**	1.040	0.243
**miR-017-5p**	1.007	0.270
**miR-331**	0.930	0.312
**miR-125a**	0.807	0.368
**miR-015b**	0.796	0.178
miR-126*	0.752	0.291
**miR-026b**	0.477	0.294
**miR-342**	0.475	0.344
**miR-628**	0.413	0.431
**miR-106a**	0.395	0.248
**miR-142-5p**	0.360	0.222
**miR-320**	0.330	0.149
**let-7b**	0.290	0.142
**miR-030a-5p**	0.271	0.141
**miR-106b**	0.247	0.185
miR-182*	0.225	0.161
**miR-374**	0.224	0.196
**miR-027a**	0.203	0.160
**miR-030d**	0.158	0.096
**miR-423**	0.124	0.096
**miR-021**	0.120	0.111
**miR-195**	0.116	0.078
**miR-133b**	0.104	0.072

Normalized expression of miRNAs in the platelets (n = 6) was calculated using median normalization analysis then the mean C_T_ value was converted to expression (2^∧(−ΔCT)^) ±standard deviation (S.D.).

## Discussion

In this study, we hypothesized that miRNAs circulate in plasma microvesicles, platelets, and PBMC of normal human volunteers in the peripheral blood. We have characterized peripheral blood miRNA patterns in healthy humans. We found significant differences in miRNA expression between plasma microvesicles, platelets, and PBMC. To date, numerous studies have established the ability for miRNAs to regulate many cellular functions. However, these studies largely imply that the miRNA resides within its host cell to elicit an effect [39]. Our data indicate that the miRNAs are also contained in plasma microvesicles that may influence homeostasis.

The defined mechanism or cellular signals that regulate microvesicle production is unknown. Some suggest that the vesicle “hijacks” the cytoplasm prior to release from the cell through membrane blebbing [23]. This paradigm suggests that packaging of the miRNAs in the microvesicle may be random. Alternatively, endosomal trafficking may modulate the formation of microvesicles [40]. Since P-bodies in the cytoplasm co-localized with miRNAs [2], [4], we are exploring whether P-bodies may regulate the transfer and sorting of miRNAs into vesicles. There are other reports of tumor cell fragments containing miRNAs in the plasma and/or serum [41], [42]. Recently Taylor and colleagues found circulating tumor microvesicles in the peripheral blood of patients with ovarian cancer but not healthy individuals [41]. These microvesicles express miRNAs found in the primary tumor.

It is conceivable that the miRNAs in the plasma microvesicles circulate to tissue targets. Further examination of the highest expressed miRNAs in plasma microvesicles (Table 1), demonstrate that many of these miRNA are predicted to regulate hematopoiesis and cellular differentiation. For instance, expression of miR-223 is predicted to influence myeloid, granulocyte and osteoclast differentiation [43]–[45]. miR-223 also appears to have a role in hematopoietic stem cell proliferation [43]. Interestingly, miR-223 expression is lost in acute myelogenous leukemia (AML) [46]. miR-24 expression is regulated by TGF-β, a potent positive and negative regulator of hematopoiesis [47], [48]. Both miR-24 and miR-16 regulate red cell production [49], [50], while miR-16 also modulates lymphoid development [51]. Loss of miR-16 expression has been extensively examined in chronic lymphocytic leukemia (CLL) [52], [53]. In contrast, downregulation of miR-126 expression occurs during megakarocyte differentiation [54].

Many miRNAs expressed in the plasma microvesicles also regulate the progression of the cell cycle proteins [55], [56]. miR-222 targets p27Kip1 [55] while miR-24 suppresses p16 (INK4a) [56]. Increased expression of miR-16 results in the accumulation of cells in G0/G1 phase of the cell cycle [47].

To date, there are no known functions for miR-484 which is the second highest expressed miRNA in the plasma microvesicles fraction based on normalized expression (Table 1). Using IPA analysis, miR-484 appears to regulate hematopoiesis, as well as cellular differentiation, proliferation and growth similar to many of the highly expressed miRNAs in the plasma microvesicles. Another of the highly expressed miRNAs, miR-146a is predicted to modulate NK cell signaling and IL-4 signaling pathways. Similarly, miR-146a is also predicted to regulate immune functions [57], [58]. Based on IPA analysis examining gene ontology of targets, the top associated networks predicted to be influenced by miR-146a expression are cellular proliferation, immune and lymphatic system development and function. In addition, this miRNA is predicted to regulate innate immune responses. From the analysis, we found that LPS/IL-1 and toll-like receptor signaling are among the top five canonical pathways predicted to be regulated by miR-146a.

Comparison of differentially expressed miRNAs between the plasma microvesicles and the PBMC indicated that miR-486 was the highest differentially expressed in the plasma microvesicles (Table 3). Little is known about this miRNA, except that it is expressed in glioblastoma cells and may function in brain cell differentiation [59]. Analyzing the predicted targets from Sanger miRBase with the IPA software, the top five canonical pathways predicted to be regulated by miR-486 are those in phenylalanine and cyanoamino acid metabolism, insulin receptor signaling, antigen presentation, and pentose phosphate pathways. Another miRNA differentially expressed in the plasma microvesicles is miR-328. Recently, Wang and colleagues reported that miR-328 is important in regulating tumor development and invasion by targeting CD44 expression in epithelial carcinoma cell lines [60]. Overexpression of miR-328 inhibits cell migration and adhesion and preventing them to expand into the tumor.

The majority of the plasma microvesicles from normal individuals are derived from blood cells. We did detect a small percentage of microvesicles derived from endothelial cells. While human endothelial progenitor cells can produce microvesicles *in vitro* [26], detection of the endothelial derived vesicles in circulation is of interest. We would predict that the endothelial-derived microvesicles may increase upon endothelial cell damage. Recently, elevation of endothelial-derived microvesicles has been reported in patients with pulmonary arterial hypertension [32], however their content is currently unknown. Likewise, the detection of tissue specific miRNAs and microvesicles in the peripheral blood may be a frequent event upon tissue damage. The detection of tumors exosomes (microvesicles) have been found in the peripheral blood to contain miRNAs [41]. Interestingly, comparing this paper's findings with our data, 18 elevated tumor-derived miRNAs were not detected in the plasma from our normal donors (Table S1).

While it has been reported that miRNAs are detected in the plasma [41], [42], [61]–[63], this is the first study to profile miRNAs from the plasma microvesicles in a normal healthy population. Since our study was limited to Caucasians, we do not know the influence of race or ethnicity. In this study, we also examined whether miRNA expression in the plasma microvesicles was influenced by age. Surprisingly, we observed no difference between gender and age in our study. These are important factors as we and others examine miRNA expression from the plasma and/or serum in various diseases to serve as diagnostic biomarkers.

Our findings indicate that miRNAs circulate in the plasma and PBMC of normal donors. These miRNAs are predicted to regulate homeostasis of hematopoietic cells and of metabolic function. Investigations are currently underway to understand if specific cells are targeted by the plasma microvesicles and determine the influence of these microvesicle-containing miRNA on target cell mRNA expression. We predict that the plasma microvesicles may be selective in their target cells. For instances, platelet-derived microparticles transfer tissue factor to monocytes but not neutrophils [64]. Thus, we predict that the defined function of each subpopulation of the plasma microvesicles and their miRNAs will be important factors in the regulation of immune responses and hematopoiesis.

## Supporting Information

Table S1 Normalized expression for plasma microvesicles and PBMC. Expression of miRNAs in the plasma microvesicles and PBMC were analyzed separately (n = 51). The normalized miRNA expression was generated using median normalization analysis then the mean CT value was converted to expression (2∧(−deltaCT)) ±standard deviation (S.D.).
(0.01 MB PDF)

Table S2 Predicted targets for miRNAs expressed in the plasma microvesicles and PBMC. Predicted targets were compiled separately using Sanger miRBase and TargetScan for the top nine miRNAs expressed in the plasma microvesicles and PBMC (Table 1). Targets were common between the databases were furthered analyzed for predicted function.
(0.20 MB PDF)

Table S3 Comparison of miRNA expression between plasma microvesicles and PBMC. In order to test the differences of miRNA expression between plasma microvesicles and PBMC, data were analyzed using linear mixed models. The p-values were generated from the model based on the estimated difference and sample variation. A bonferroni adjustment for multiple comparisons (72 comparisons) to control type I error to reduce the number of false positives was performed, therefore “p-value” was considered as significant if p-value<0.05/72 = 0.0006. Fold-change was calculated based on the estimated mean difference (2∧(−ΔCT)).
(0.01 MB PDF)
