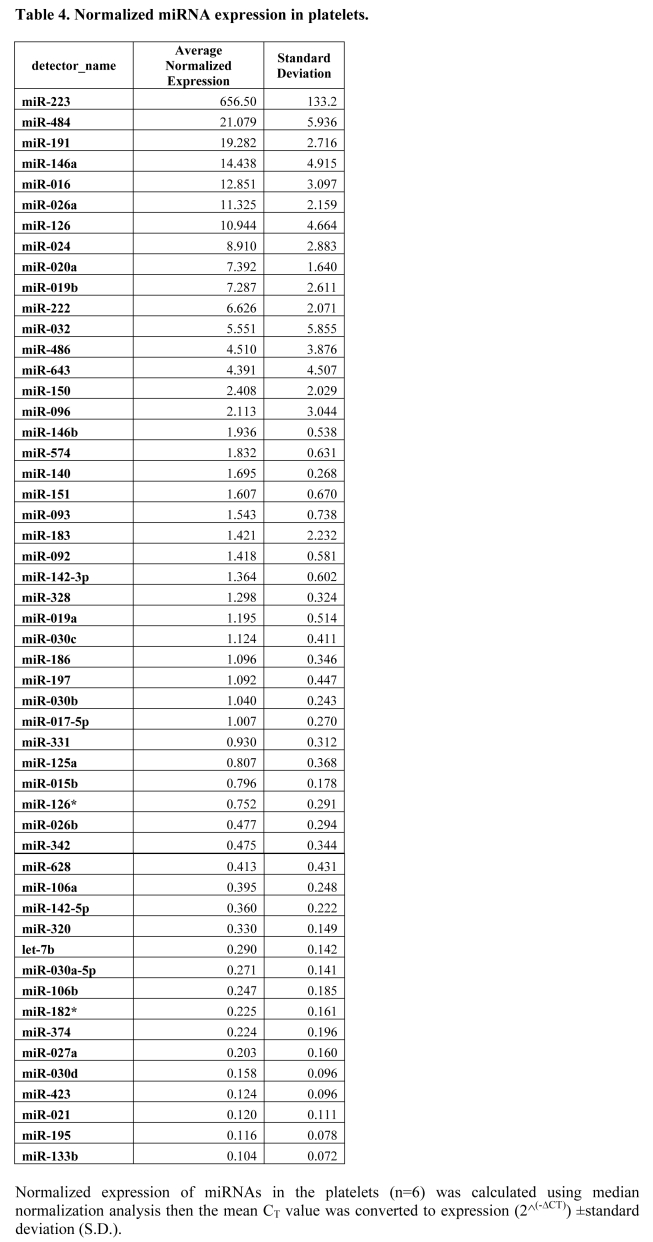# Correction: Detection of microRNA Expression in Human Peripheral Blood Microvesicles

**DOI:** 10.1371/annotation/b15ca816-7b62-4474-a568-6b60b8959742

**Published:** 2010-03-09

**Authors:** Melissa Piper Hunter, Noura Ismail, Xiaoli Zhang, Baltazar D. Aguda, Eun Joo Lee, Lianbo Yu, Tao Xiao, Jeffrey Schafer, Mei-Ling Ting Lee, Thomas D. Schmittgen, S. Patrick Nana-Sinkam, David Jarjoura, Clay B. Marsh

A line is missing from Table 4. Please view the correct table at: 

**Figure:**