# Supplementary material for: Detection of microRNA Expression in Human Peripheral Blood Microvesicles
Source: PLoS One. 2008 Nov 11;3(11):e3694. doi: 10.1371/journal.pone.0003694 (PMC2577891; doi:10.1371/journal.pone.0003694)
Supplement: Table S1 — Normalized expression for plasma microvesicles and PBMC. Expression of miRNAs in the plasma microvesicles and PBMC were analyzed separately (n = 51). The normalized miRNA expression was generated using median normalization analysis then the mean CT value was converted to expression (2∧(−deltaCT)) ±standard deviation (S.D.). (0.01 MB PDF) [file pone.0003694.s001.pdf]

Plasma Microvesicles			PBMC		
detector_name	Average Normalized Expression	Standard Deviation	detector_name	Average Normalized Expression	Standard Deviation
hsa-miR-223	1589.265353	653.1441016	hsa-miR-223	2143.797514	499.6723532
hsa-miR-484	50.93154102	22.91410685	hsa-miR-150	241.3339986	94.6316509
hsa-miR-191	46.44422571	14.98219093	hsa-miR-146b	57.51515588	21.17347322
hsa-miR-146a	39.5631478	19.020171	hsa-miR-016	54.79023342	32.92864759
hsa-miR-016	25.45043823	13.32434705	hsa-miR-484	40.62828652	18.89513879
hsa-miR-026a	25.2068328	9.956681628	hsa-miR-146a	39.66536453	13.01422255
hsa-miR-222	24.51626706	12.42177425	hsa-miR-191	32.44104295	15.62493155
hsa-miR-024	22.79169634	10.53596037	hsa-miR-026a	30.05789737	8.928309461
hsa-miR-126	18.20923598	8.046659812	hsa-miR-019b	21.72111133	7.496587814
hsa-miR-032	15.32305403	32.61653539	hsa-miR-020a	15.03972998	5.117918017
hsa-miR-486	12.89548349	11.97427815	hsa-miR-024	14.49037987	2.924884427
hsa-miR-020a	10.12067658	5.597534469	hsa-miR-142-3p	11.61802275	7.535807778
hsa-miR-019b	9.158209689	6.764220029	hsa-miR-140	11.15286241	3.933184767
hsa-miR-150	8.165508668	25.08223076	hsa-miR-126	9.209558485	3.825687483
hsa-miR-574	5.939301367	3.72201371	hsa-miR-342	7.803100549	3.388772813
hsa-miR-092	5.700980896	3.157407121	hsa-miR-155	6.584151232	4.579272166
hsa-miR-093	5.436799962	2.417329407	hsa-miR-222	5.715723219	2.105344287
hsa-miR-342	5.114543492	3.297304604	hsa-miR-019a	5.53562668	2.957936571
hsa-miR-197	4.9808586	4.507000203	hsa-miR-093	3.756090874	1.000518353
hsa-miR-328	4.707858075	3.59258757	hsa-miR-092	3.215354275	0.85390195
hsa-miR-096	3.674085488	5.965291968	hsa-miR-486	3.096228853	7.676887526
hsa-miR-151	3.452172299	1.083769726	hsa-miR-030b	2.865611791	0.76538861
hsa-miR-146b	3.272000368	2.10149536	hsa-miR-574	2.458205492	1.894539696
hsa-miR-140	3.196516876	1.716779701	hsa-miR-030c	2.335820956	0.663949738
hsa-miR-030b	3.134519745	1.142809477	hsa-miR-026b	2.129402092	0.705755408
hsa-miR-125a	3.080410769	1.975408888	hsa-miR-331	1.985929854	0.609153141
hsa-miR-126*	3.023376204	2.068205543	hsa-miR-125a	1.808127477	1.087896802
hsa-miR-183	2.854264163	6.494415425	hsa-miR-186	1.720577641	0.419612713
hsa-miR-030c	2.412532336	0.863586828	hsa-miR-032	1.716467503	1.338128537
hsa-miR-017-5p	2.13024101	0.998316036	hsa-miR-029a	1.323099465	0.533548819
hsa-miR-331	2.087433557	0.812550081	hsa-miR-126*	1.269368187	0.759421276
hsa-miR-186	2.022411766	0.916917175	hsa-let-7g	1.199246639	0.349725473
hsa-miR-015b	1.808314178	0.810480106	hsa-miR-021	1.161213483	0.674584117
hsa-miR-019a	1.697172987	1.453896733	hsa-miR-197	1.149407136	0.647186919
hsa-miR-302b	1.339313335	2.387043602	hsa-miR-015b	1.14628446	0.432407565
hsa-miR-026b	1.292369054	0.824157842	hsa-miR-030a-5p	1.094683149	0.306485271
hsa-miR-106a	1.186157531	1.293289083	hsa-miR-195	1.017673959	0.797387672
hsa-let-7b	0.97666637	0.625535748	hsa-miR-151	1.007960478	0.397967603
hsa-miR-320	0.911092198	0.420502686	hsa-miR-142-5p	0.962665368	0.492490733
hsa-miR-155	0.808867743	0.52866023	hsa-miR-017-5p	0.941159371	0.360846484
hsa-miR-030a-5p	0.727538734	0.549256565	hsa-miR-106b	0.910164376	0.403715009
hsa-miR-628	0.713396354	1.263218707	hsa-miR-096	0.875989336	0.559638262
hsa-miR-027a	0.641903058	0.448916565	hsa-miR-374	0.764917256	0.274388929
hsa-miR-142-3p	0.627217683	0.607184284	hsa-miR-328	0.720096352	0.412158586
hsa-miR-195	0.564628001	0.392046259	hsa-miR-030d	0.662654685	0.22361572
hsa-miR-425-5p	0.554428361	0.275865472	hsa-miR-027a	0.622821419	0.251240619
hsa-let-7g	0.515997707	0.337363561	hsa-miR-106a	0.542577626	0.312616405
hsa-miR-021	0.51383279	0.491007765	hsa-let-7b	0.534079686	0.25068168
hsa-miR-199a*	0.503454664	0.327551326	hsa-miR-020b	0.410435233	0.262371622
hsa-miR-142-5p	0.485752905	0.504503986	hsa-miR-320	0.344918355	0.140441973
hsa-miR-103	0.469571039	0.21589537	hsa-miR-425-5p	0.334114444	0.131686276
hsa-miR-106b	0.455236625	0.295268175	hsa-miR-628	0.323074686	0.328272174
hsa-miR-182*	0.450866408	0.77963697	hsa-miR-302b	0.31107497	0.233922375
hsa-miR-196b	0.424682779	0.283317957	hsa-miR-532	0.30283697	0.175299492
hsa-miR-643	0.410079866	0.934728725	hsa-miR-196b	0.291839926	0.203868959
hsa-miR-030d	0.360317322	0.195418907	hsa-miR-015a	0.263528933	0.156658681

Plasma Microvesicles			PBMC		
**detector_name**	**Average Normalized Expression**	**Standard Deviation**	**detector_name**	**Average Normalized Expression**	**Standard Deviation**
**hsa-miR-423**	0.355300524	0.267033256	**hsa-miR-183**	0.223615225	0.225419033
**hsa-let-7a**	0.316043353	0.197156608	**hsa-miR-345**	0.222589939	0.14617654
**hsa-miR-027b**	0.252490316	0.182518193	**hsa-miR-423**	0.213547231	0.136842846
**hsa-miR-374**	0.251770179	0.197212133	**hsa-miR-103**	0.205412359	0.093491856
**hsa-miR-020b**	0.227198431	0.167978797	**hsa-let-7a**	0.198065472	0.082808812
**hsa-miR-133b**	0.201227753	0.178371953	**hsa-miR-181d**	0.170055022	0.109234879
**hsa-miR-425-3p**	0.183808363	0.094868402	**hsa-miR-182***	0.151155794	0.079789533
**hsa-miR-376a**	0.166829158	0.161986415	**hsa-miR-340**	0.128492001	0.08303611
**hsa-miR-340**	0.113550436	0.088995419	**hsa-miR-425-3p**	0.127886265	0.097067479
**hsa-miR-015a**	0.110336695	0.095621341	**hsa-miR-199a***	0.127527328	0.079775882
**hsa-miR-181d**	0.093074354	0.212992595	**hsa-miR-376a**	0.124903907	0.160137603
**hsa-miR-532**	0.086633253	0.064244146	**hsa-miR-643**	0.116164417	0.195641411
**hsa-miR-025**	0.072911126	0.043856514	**hsa-miR-025**	0.11319355	0.079644761
**hsa-miR-345**	0.071420142	0.054898774	**hsa-miR-133b**	0.100108157	0.121551814
**hsa-miR-029a**	0.058322216	0.093625329	**hsa-miR-027b**	0.07101679	0.067406886
