# Supplementary material for: Detection of microRNA Expression in Human Peripheral Blood Microvesicles
Source: PLoS One. 2008 Nov 11;3(11):e3694. doi: 10.1371/journal.pone.0003694 (PMC2577891; doi:10.1371/journal.pone.0003694)
Supplement: Table S2 — Predicted targets for miRNAs expressed in the plasma microvesicles and PBMC. Predicted targets were compiled separately using Sanger miRBase and TargetScan for the top nine miRNAs expressed in the plasma microvesicles and PBMC (Table 1). Targets were common between the databases were furthered analyzed for predicted function. (0.20 MB PDF) [file pone.0003694.s002.pdf]

Plasma Microvesicles Predicted Targets			PBMC Predicted Targets		
Sanger miRBase	TargetScan	Common	Sanger miRBase	TargetScan	Common
A1A4G5_HUMAN	AADACL1	AARS	A1A5D9_HUMAN	AADACL1	ABCB7
A1L162_HUMAN	AARS	ABCB9	A1A5D9_HUMAN	ABCA1	ABHD2
A2A3T8_HUMAN	ABCA1	ABHD2	A1L162_HUMAN	ABCB7	ACBD5
A4D193_HUMAN	ABCB9	ACBD5	A4D193_HUMAN	ABCC4	AP2A1
AADAC	ABCC4	AP2A1	AANAT	ABCC5	ARMCX2
AARS	ABCC5	ARMCX2	AARS	ABCG4	ASNA1
AATF	ABCG4	C17orf49	ABCA1	ABHD13	B3GALNT2
ABCA1	ABHD13	C20orf24	ABCA1	ABHD2	C20orf24
ABCA4	ABHD13	CASK	ABCA4	ABR	C2orf42
ABCA4	ABHD2	CCNE1	ABCA4	ACADSB	CASK
ABCB8	ABHD2	CCNT2	ABCB7	ACBD3	CCNE1
ABCB9	ACBD4	CCT3	ABCB7	ACBD5	CCNT2
ABCC11	ACBD5	CD164L2	ABCC11	ACBD5	CHEK1
ABCD4	ACOX1	CHD5	ABCD4	ACCN2	CHPT1
ABCF2	ACSBG1	CHEK1	ABCF2	ACCN4	CRTC2
ABHD12	ACSL3	CHPT1	ABHD2	ACOX1	DLGAP1
ABHD12	ACSL3	CRTC2	ABHD7	ACSBG1	EDA
ABHD2	ACSL4	CSK	ABHD8	ACSL1	ELOVL3
ABHD8	ACTA1	DLGAP1	ABI2	ACSL3	ENPP5
ABI2	ACTR2	EPHA1	ACBD5	ACSL4	EPHA1
ACAD9	ACTR3B	EZH2	ACO1	ACSL4	EZH2
ACBD5	ACVR1B	FBXO8	ACOT7	ACTA1	FASTK
ACE	ACVR1B	FREM2	ACOX3	ACTN1	FBXO8
ACOT7	ACVR1C	FSD1	ACOX3	ACTR1A	FBXO8
ACOX3	ACVR2A	GAL3ST3	ACOX3	ACTR2	FSD1
ACOX3	ACVR2A	GBA2	ACOX3	ACTR3B	GLUD2
ACOX3	ACVR2B	GLUD2	ACRC	ACVR1B	GPR63
ACOX3	ACVR2B	GPR63	ACRC	ACVR1C	GRIA3
ACOX3	ACVR2B	GRIA3	ACTA1	ACVR2A	HD
ACRC	ADAM10	H2AFX	ACTA1	ACVR2B	HDGF
ACRC	ADAM11	HD	ACTA1	ADAM10	HECTD3
ACSL6	ADAM19	HDGF	ACTRT2	ADAM11	HELZ
ACSL6	ADAMTS18	HECTD3	ACTRT2	ADAM12	HIPK3
ACSL6	ADAMTS19	HELZ	ADAM22	ADAM19	HOXA5
ACSL6	ADAMTS3	HOXA5	ADAM22	ADAMTS18	HTR4
ACSL6	ADAMTS3	HTR4	ADH6	ADAMTS19	IMPDH1
ACTA1	ADAMTS5	INPP5B	ADMR	ADAMTS3	INPP5B
ACTA1	ADAMTS6	INTU	ADRA1A	ADAMTS5	INTU
ACTA1	ADAMTSL3	KCNQ4	ADRA1A	ADAMTS6	JAKMIP1
ADAM22	ADARB1	KIAA0241	AGPAT4	ADAMTSL3	KCNQ4
ADAMTSL4	ADM	KIAA0241	AGR3	ADARB1	KIAA0241
AGPAT2	ADRB2	KIF21A	AGXT2L2	ADCY1	KIF21A
AGPAT4	AFF2	LELP1	AHI1	ADCY7	LELP1
AGXT2L2	AFF4	LRFN2	AHI1	ADCY9	LRP2
AHI1	AGPAT3	LYPLA2	AHSA1	ADD3	MAN2A1
AHI1	AK3L1	MAN2A1	AIM1L	ADIPOR2	MBD6
AHSA1	AKAP11	MAP3K10	AJAP1	ADM	MIER1
AJAP1	AKAP7	MBOAT1	AKAP9	ADNP	NME7
AK1	AKT3	MESDC1	AKR1C3	ADRB1	NOL4
AKAP9	ALCAM	MTMR14	ALB	ADRB2	OMG
ALAS2	ALDH5A1	NAPRT1	ALB	ADRBK1	PDHX
ALB	ALG2	NOL4	ALG12	ADSS	PLCD1
ALB	ALS2	NSMCE4A	ALKBH3	AEBP2	PLXNC1
ALG12	ALS2CR2	OMG	ALKBH3	AFF1	PPAP2A
ALKBH3	ALS2CR2	PDHX	ALMS1	AFF2	PRKCD

Plasma Microvesicles Predicted Targets			PBMC Predicted Targets		
**Sanger miRBase**	**TargetScan**	**Common**	**Sanger miRBase**	**TargetScan**	**Common**
ALKBH3	ALS2CR2	PLCD1	ALPK2	AFF4	PTH
ALS2CR12	AMMECR1	PPAP2A	ALS2CR12	AFTPH	RAB21
ANKMY1	AMMECR1L	PRKCD	ALS2CR12	AK3L1	RAP1A
ANKRD44	AMMECR1L	PTH	ALS2CR12	AKAP1	RBM6
ANTXRL	AMOT	RBM6	AMAC1L1	AKAP11	SCN3A
ANXA9	AMOTL1	RHBDL1	AMAC1L3	AKAP6	SCN8A
ANXA9	AMOTL2	SCN3A	ANK1	AKAP7	SCOC
AP2A1	ANGEL1	SCN8A	ANKRD44	AKT3	SEMA4C
AP2S1	ANGEL1	SCOC	ANKRD58	ALCAM	SEMA6D
AP3B2	ANGPTL2	SEMA6D	ANKS6	ALDH5A1	SENP5
APEX1	ANK2	SENP5	ANKZF1	ALS2	SGMS2
APOBEC3D	ANK2	SGMS2	ANPEP	ALS2CR2	SIAH1
APOD	ANK2	SIAH1	ANTXRL	AMMECR1	SLC25A35
APOM	ANKHD1	SLC12A6	ANXA9	AMMECR1L	SLC27A2
APTX	ANKRD15	SLC25A35	ANXA9	AMOT	SNIP
APTX	ANKRD16	SLC25A37	ANXA9	AMOTL1	SRP19
APTX	ANKRD17	SRP19	ANXA9	AMOTL2	STK19
AQP11	ANKRD17	STK19	AP1G2	AMPH	STK33
ARAF	ANKRD57	STK33	AP2A1	ANGEL1	STMN1
ARAF	ANKS1A	STMN1	AP2A1	ANK2	STOX2
ARD1A	ANKS1B	STOX2	APEX1	ANKRD11	STRBP
ARD1A	AP1G1	STRBP	APOBEC3D	ANKRD13B	TAF12
ARID1A	AP1GBP1	SUV420H2	APOD	ANKRD15	TAF15
ARMC4	AP2A1	TAF12	APOM	ANKRD16	TBC1D10A
ARMCX2	APBA1	TAF15	APRT	ANKRD17	TBP
ARMCX2	APC	TBC1D10A	APTX	ANKRD57	TGIF1
ARMCX5	APLN	TBP	APTX	ANKS1A	TMEM93
ARMCX5	APOA5	TCF7	AQP11	ANKS1B	TRAF6
ARPC3	APP	TMEM93	ARAF	ANXA7	TSPYL2
ARPC5	APPL2	TRAF6	ARAF	AP1G1	USP15
ARSA	APRIN	TRIM9	ARAF	AP1GBP1	ZDHHC18
ART3	ARCN1	TSPAN14	ARD1A	AP2A1	ZDHHC6
ART3	ARF4	TSPYL2	ARD1A	APC	ZIC5
ART3	ARF4	UBQLNL	ARD1A	APCDD1	ZYX
ASB1	ARHGAP12	USP15	ARF5	APLN	
ASB2	ARHGAP17	ZDHHC18	ARF5	APP	
ASB4	ARHGAP20	ZDHHC6	ARHGAP29	APPL1	
ATAD3B	ARHGAP21	ZIC5	ARID1A	APPL2	
ATAD3B	ARHGAP26	ZYX	ARIH2	APRIN	
ATF1	ARHGAP5		ARMCX2	ARC	
ATN1	ARHGEF7		ARMCX2	ARCN1	
ATOX1	ARHGEF9		ARMCX5	ARF1	
ATP1A1	ARID1A		ARMCX5	ARF4	
ATP5C1	ARID2		ARPC2	ARF5	
ATP5C1	ARID2		ARPC3	ARFGEF1	
ATP5E	ARID3A		ARPC5	ARFIP1	
ATP6AP1	ARID5B		ARRB2	ARHGAP1	
ATP8A1	ARL10		ARRB2	ARHGAP12	
ATXN7L2	ARL2		ARSA	ARHGAP17	
AZI2	ARL4C		ART3	ARHGAP20	
B3GAT3	ARL6IP1		ART3	ARHGAP21	
B3GNT1	ARL6IP6		ART3	ARHGAP26	
B3GNT4	ARMCX2		ASB1	ARHGAP5	
B4GALNT4	ARMCX2		ASB2	ARHGDIA	
B4GALNT4	ARNT		ASB4	ARHGEF10L	

Plasma Microvesicles Predicted Targets			PBMC Predicted Targets		
Sanger miRBase	TargetScan	Common	Sanger miRBase	TargetScan	Common
BAI3	ARPP-19		ASNA1	ARHGEF12	
BAT4	ARPP-21		ASXL1	ARHGEF9	
BAT4	ASB7		ATF1	ARID1A	
BAT4	ASH1L		ATG16L2	ARID2	
BAT4	ASPH		ATG16L2	ARID3A	
BAT4	ASPN		ATOX1	ARID4B	
BAT4	ATBF1		ATP5C1	ARID5B	
BAT4	ATBF1		ATP5C1	ARIH2	
BAT4	ATF2		ATP5E	ARL10	
BAT5_HUMAN	ATF7IP2		ATP6AP1	ARL2	
BBS1	ATP11C		ATP8A1	ARL4C	
BCAP31	ATP13A2		ATXN7L2	ARL6IP1	
BCAP31	ATP1A2		AZI2	ARL6IP2	
BCAR1	ATP1B2		B3GALNT2	ARL6IP6	
BCL7B	ATP1B4		B3GALNT2	ARL8B	
BCOR	ATP2B1		B3GNT1	ARMC5	
BCOR	ATP2B2		B3GNT4	ARMC8	
BCOR	ATP2B2		B4GALNT4	ARMCX2	
BEST3	ATP2C1		B4GALNT4	ARPP-19	
BID	ATP6V0A2		BAI1	ARPP-21	
BLVRA	ATP7A		BAI3	ARRDC3	
BLZF1	ATPAF1		BARD1	ARRDC4	
BLZF1	ATXN1		BASP1	ARVCF	
BLZF1	ATXN2		BAT4	ASH1L	
BMP1	AXIN2		BAT4	ASNA1	
BMP1	AXIN2		BAT4	ASPH	
BNIP1	AZI2		BAT4	ASPN	
BNIP1	B3GNT1		BAT5_HUMAN	ASXL1	
BOLA3	B4GALT1		BAX	ASXL2	
BOLL	B4GALT1		BAX	ATBF1	
BRUNOL5	BAAT		BAX	ATF2	
BSND	BACE1		BBS1	ATF7IP2	
BVES	BACH2		BCAP31	ATG16L1	
BXDC5	BAG4		BCAR1	ATP10A	
C10orf110	BAG5		BCL2L14	ATP11A	
C10orf113	BAI3		BCL3	ATP11C	
C10orf129	BAK1		BCL7B	ATP1A2	
C10orf129	BAZ2B		BCOR	ATP1B2	
C10orf132	BAZ2B		BCOR	ATP1B4	
C10orf65	BCL11A		BEST3	ATP2B1	
C10orf82	BCL2L11		BID	ATP2B2	
C10orf82	BCL2L11		BLZF1	ATP2C1	
C10orf92	BCL2L2		BLZF1	ATP6V0C	
C10orf97	BCORL1		BLZF1	ATP6V1B2	
C11orf59	BCR		BLZF1	ATP7A	
C11orf77	BDNF		BMP1	ATP8A2	
C12orf24	BDNF		BMP1	ATPAF1	
C12orf50	BFAR		BOLA3	ATRX	
C13orf15	BHLHB2		BRUNOL5	ATXN1	
C13orf18	BHLHB3		BRUNOL5	ATXN2	
C13orf18	BIVM		BSND	AUH	
C13orf18	BLOC1S2		BSND	AXIN2	
C14orf54	BMF		BST1	AZI2	
C14orf54	BMPR1A		BST1	B3GALNT2	
C16orf42	BMX		BXDC5	B4GALT1	

Plasma Microvesicles Predicted Targets			PBMC Predicted Targets		
Sanger miRBase	TargetScan	Common	Sanger miRBase	TargetScan	Common
C16orf53	BRMS1L		BXDC5	B4GALT2	
C16orf53	BRWD1		C10orf110	B4GALT5	
C17orf49	BTAF1		C10orf113	BAAT	
C17orf60	BTBD7		C10orf129	BACE1	
C17orf64	BTG1		C10orf132	BACH2	
C18orf56	BTG2		C10orf132	BAG4	
C19orf15	BTG4		C10orf28	BAG5	
C19orf15	BTRC		C10orf28	BAI1	
C19orf18	BZW1		C10orf47	BAI3	
C19orf18	C10orf12		C10orf47	BAK1	
C19orf28	C10orf137		C10orf80	BAMBI	
C19orf36	C10orf22		C10orf92	BASP1	
C19orf50	C10orf46		C10orf93	BAZ2A	
C19orf56	C10orf54		C10orf97	BAZ2B	
C1orf102	C10orf56		C10orf99	BBX	
C1orf102	C10orf56		C11orf36	BCAT2	
C1orf102	C11orf30		C11orf59	BCL11A	
C1orf145	C11orf68		C11orf77	BCL2	
C1orf148	C12orf23		C12orf50	BCL2L11	
C1orf186	C12orf30		C13orf15	BCL2L2	
C1orf186	C12orf30		C14orf149	BCL3	
C1orf34	C12orf35		C14orf37	BCL6	
C1orf53	C12orf48		C14orf54	BCL9L	
C1orf61	C12orf64		C14orf54	BCORL1	
C1orf61	C13orf18		C15orf24	BCR	
C1orf61	C14orf129		C16orf53	BDNF	
C1orf65	C14orf32		C17orf64	BET1	
C1orf65	C16orf69		C18orf16	BFAR	
C1orf88	C16orf72		C19orf15	BHLHB2	
C1orf94	C17orf49		C19orf15	BHLHB3	
C20orf133	C17orf59		C19orf18	BICD2	
C20orf133	C18orf25		C19orf24	BIVM	
C20orf133	C18orf25		C19orf28	BLCAP	
C20orf24	C1orf108		C19orf52	BLOC1S2	
C20orf58	C1orf21		C19orf56	BMPER	
C20orf58	C1orf9		C1orf102	BMPR1A	
C20orf58	C1QL3		C1orf102	BMPR2	
C20orf80	C20orf23		C1orf102	BMX	
C20orf80	C20orf23		C1orf120	BNC2	
C20orf86	C20orf24		C1orf133	BOLA2	
C20orf96	C20orf39		C1orf145	BPTF	
C20orf96	C20orf46		C1orf145	BRMS1L	
C21orf34	C22orf5		C1orf148	BRWD1	
C21orf70	C2orf43		C1orf170	BSN	
C2orf30	C3orf23		C1orf177	BTAF1	
C2orf30	C5orf23		C1orf177	BTBD7	
C2orf42	C6orf213		C1orf186	BTF3L4	
C3orf24	C6orf85		C1orf186	BTG1	
C3orf24	C7orf42		C1orf186	BTG2	
C3orf28	C8orf46		C1orf186	BTG4	
C4orf12	C8orf58		C1orf43	BTRC	
C5orf26	C9orf150		C1orf43	BZRAP1	
C5orf26	C9orf39		C1orf43	C10orf12	
C5orf34	C9orf40		C1orf53	C10orf137	
C5orf37	C9orf5		C1orf61	C10orf46	

Plasma Microvesicles Predicted Targets			PBMC Predicted Targets		
Sanger miRBase	TargetScan	Common	Sanger miRBase	TargetScan	Common
C6orf111	CACHD1		C1orf61	C10orf54	
C6orf130	CACNA1C		C1orf61	C10orf56	
C6orf130	CACNB1		C1orf85	C11orf30	
C6orf173	CACNB2		C1orf88	C11orf68	
C6orf25	CADM1		C1orf94	C12orf23	
C7orf28B	CALCR		C20orf12	C12orf30	
C8orf14	CALM1		C20orf133	C12orf35	
C8orf70	CALML4		C20orf133	C12orf48	
C9orf130	CAMKK1		C20orf133	C12orf53	
C9orf130	CAMSAP1		C20orf134	C12orf64	
C9orf130	CAMSAP1		C20orf24	C13orf18	
C9orf135	CAMSAP1		C20orf46	C14orf101	
C9orf138	CAMSAP1L1		C20orf46	C14orf129	
C9orf139	CAPN6		C20orf58	C14orf147	
C9orf142	CAPZA1		C20orf58	C14orf32	
C9orf16	CAPZA2		C20orf58	C14orf4	
C9orf27	CAPZB		C20orf58	C16orf14	
C9orf66	CARM1		C20orf80	C16orf69	
C9orf86	CASK		C20orf80	C16orf70	
CABYR	CASP8AP2		C20orf96	C16orf72	
CACNA1D	CASR		C21orf34	C17orf39	
CALCA	CASZ1		C21orf70	C17orf59	
CASK	CASZ1		C2orf24	C18orf25	
CBARA1	CBFA2T3		C2orf30	C1orf108	
CBLC	CBFB		C2orf30	C1orf121	
CCDC103	CBL		C2orf42	C1orf21	
CCDC110	CBX4		C2orf42	C1orf9	
CCDC114	CC2D1B		C3orf24	C1orf96	
CCDC128	CCDC117		C3orf24	C1QL3	
CCDC129	CCDC131		C3orf24	C20orf174	
CCDC18	CCDC28A		C3orf24	C20orf23	
CCDC18	CCDC28A		C3orf42	C20orf24	
CCDC53	CCDC58		C5orf26	C20orf39	
CCDC74A	CCDC6		C5orf26	C20orf46	
CCDC74B	CCDC6		C5orf34	C22orf5	
CCDC76	CCDC64		C5orf37	C22orf9	
CCDC96	CCND1		C6orf111	C2orf17	
CCK	CCND2		C6orf128	C2orf42	
CCKBR	CCND2		C6orf173	C3orf23	
CCL13	CCNE1		C6orf203	C3orf59	
CCL18	CCNE2		C6orf25	C4orf31	
CCL3	CCNI		C7orf28B	C5orf23	
CCL3L1	CCNJ		C8orf70	C5orf30	
CCL3L3	CCNJ		C9orf103	C6orf134	
CCL4	CCNJL		C9orf103	C6orf154	
CCL4L1	CCNJL		C9orf103	C6orf49	
CCNA2	CCNT2		C9orf117	C6orf85	
CCNB3	CCNT2		C9orf138	C7orf42	
CCNB3	CCNYL1		C9orf139	C7orf43	
CCNE1	CCT3		C9orf156	C8orf58	
CCNT2	CD164		C9orf86	C9orf150	
CCNT2	CD164		CACNA1D	C9orf40	
CCR9	CD164L2		CACNA1G	C9orf5	
CCT3	CD200		CACNA2D1	CA8	
CCT6B	CD28		CALCA	CAB39	

Plasma Microvesicles Predicted Targets			PBMC Predicted Targets		
Sanger miRBase	TargetScan	Common	Sanger miRBase	TargetScan	Common
CD164L2	CD2AP		CAMK1	CACHD1	
CD1B	CD2AP		CARD10	CACNA1C	
CD244	CD2AP		CARD10	CACNA1G	
CD274	CD4		CASK	CACNB1	
CD74	CDC25A		CASK	CACNB2	
CD81	CDC27		CASZ1	CACNB3	
CD81	CDC2L5		CASZ1	CADM1	
CD99	CDC2L6		CBARA1	CADM4	
CD99	CDC37L1		CBLC	CALM1	
CDC34	CDC42EP2		CBR1	CALML4	
CDC34	CDCA4		CCDC101	CAMK2A	
CDC40	CDH11		CCDC103	CAMKV	
CDCA7L	CDH12		CCDC107	CAMSAP1	
CDK2AP2	CDH2		CCDC107	CAMSAP1L1	
CDK3	CDH2		CCDC110	CAMTA1	
CDK5	CDH4		CCDC22	CAPN6	
CDKN1C	CDK2AP1		CCDC53	CAPZA1	
CDKN3	CDK5R1		CCDC58	CAPZA2	
CDS1	CDK6		CCDC74A	CAPZB	
CDY1	CDKN1B		CCDC74B	CARD10	
CDY1	CDKN1B		CCDC76	CARM1	
CDY1	CDKN1C		CCDC88	CASK	
CEACAM21	CDKN2AIP		CCDC88	CASKIN1	
CEACAM21	CDS2		CCDC96	CASR	
CEACAM3	CDV3		CCK	CAST	
CEACAM4	CDV3		CCK	CASZ1	
CECR5	CDV3		CCKBR	CBARA1	
CECR5	CDX2		CCL13	CBFA2T3	
CENPA	CDX2		CCL18	CBFB	
CEP290	CEBPB		CCL3	CBL	
CEP63	CEBPG		CCL3L1	CBLN2	
CEP72	CECR6		CCL3L3	CBX1	
CFH	CELSR1		CCL4	CBX4	
CFH	CENPB		CCL4L1	CBX7	
CFHR1	CEP350		CCNA2	CC2D1A	
CFHR1	CGI-38		CCNA2	CC2D1B	
CFLAR	CHAC1		CCNB3	CCDC117	
CG38_HUMAN	CHAC1		CCNB3	CCDC126	
CHCHD6	CHD2		CCNB3	CCDC131	
CHD1L	CHD5		CCNE1	CCDC28A	
CHD1L	CHD7		CCNT2	CCDC47	
CHD5	CHD8		CCNT2	CCDC6	
CHEK1	CHD8		CCR9	CCDC64	
CHEK2	CHEK1		CCS	CCDC95	
CHKB	CHFR		CD160	CCM2	
CHPT1	CHFR		CD164L2	CCND1	
CHRD	CHORDC1		CD1B	CCND2	
CHRNA4	CHORDC1		CD274	CCNE1	
CHRNB3	CHPT1		CD300LB	CCNE2	
CHUK	CHRNE		CD40LG	CCNJ	
CIDEA	CHSY1		CD55	CCNJL	
CKS2	CHSY1		CD79B	CCNL1	
CLASP2	CITED4		CD81	CCNT2	
CLDN15	CLASP2		CD81	CCNYL1	
CLDN4	CLCN3		CD81	CCPG1	

Plasma Microvesicles Predicted Targets			PBMC Predicted Targets		
Sanger miRBase	TargetScan	Common	Sanger miRBase	TargetScan	Common
CLDN6	CLCN5		CD99	CCRN4L	
CLDN8	CLCN6		CD99	CD164	
CLDN9	CLDN12		CDC34	CD200	
CLDND2	CLDN2		CDC40	CD28	
CLEC4F	CLDND1		CDK3	CD2AP	
CLLU1OS	CLDND1		CDKN1C	CD69	
CLMN	CLIC5		CDKN3	CD79B	
CLSPN	CLOCK		CDKN3	CDC25A	
CLTB	CLTC		CDS1	CDC27	
CMTM1	CMPK		CDS1	CDC2L5	
CMTM5	CMPK		CDY1	CDC2L6	
CNGA1	CMTM4		CDY1	CDC37L1	
CNGB1	CMTM4		CEACAM21	CDC42BPA	
CNGB1	CNOT2		CEACAM3	CDC42EP2	
CNTFR	CNOT2		CEACAM4	CDCA4	
CNTNAP3	CNOT6		CECR5	CDH11	
COL9A2	CNOT6L		CECR5	CDH12	
COPS4	CNTNAP1		CENPA	CDH2	
CPO	COBLL1		CEP290	CDH4	
CRADD	COL10A1		CEP290	CDK2AP1	
CRADD	COL11A1		CEP63	CDK5R1	
CREB3L3	COL11A2		CEP72	CDK6	
CRH	COL12A1		CFH	CDR2	
CRIPAK	COL12A1		CFH	CDR2L	
CRKL	COL19A1		CFH	CDS2	
CRLF1	COL1A2		CFH	CDV3	
CRP	COL24A1		CFHR1	CDX2	
CRP	COL5A1		CFHR1	CEBPB	
CRTC2	COPS2		CFHR1	CEBPG	
CRTC2	COPS2		CFHR1	CECR6	
CSF1	COPS2		CFLAR	CELSR1	
CSK	COPS7B		CG38_HUMAN	CENPB	
CSMD1	COPS7B		CHCHD6	CENTD3	
CSPG5	COX5A		CHD1L	CENTG3	
CST9L	CPD		CHD1L	CEP170	
CST9L	CPEB2		CHD1L	CEP350	
CTNNAL1	CPEB3		CHD1L	CGI-38	
CTNNAL1	CPNE8		CHD5	CGN	
CTNNAL1	CPSF2		CHEK1	CHAC1	
CTRL	CREB3L2		CHEK2	CHD2	
CTRL	CREBBP		CHGA	CHD5	
CTSA	CREBL2		CHGA	CHD6	
CTSA	CREBL2		CHPT1	CHD8	
CTSD	CREBZF		CHRNA2	CHD9	
CTSD	CREBZF		CHRNA4	CHEK1	
CTSD	CRHBP		CHRNA4	CHFR	
CTSD	CRIM1		CHRNB3	CHIC1	
CTSL1	CRIM1		CKAP4	CHMP4B	
CTSL2	CRK		CKS2	CHORDC1	
CTSL2	CRKL		CLASP2	CHPT1	
CUL4B	CRSP7		CLDN15	CHRNE	
CUL4B	CRTC2		CLDN6	CHST1	
CUTA	CRTC3		CLDN8	CHST2	
CUTA	CSDE1		CLEC4C	CHSY1	
CX04A_HUMAN	CSNK1G1		CLEC4F	CLASP2	

Plasma Microvesicles Predicted Targets			PBMC Predicted Targets		
Sanger miRBase	TargetScan	Common	Sanger miRBase	TargetScan	Common
CXCL3	CSNK1G1		CLIC1	CLCN5	
CXorf26	CTCF		CLK2	CLDN12	
CXorf26	CTDSP2		CLK2	CLDN2	
CXorf27	CTNNBIP1		CLK2	CLDND1	
CXorf59	CTNND2		CLLU1OS	CLIC5	
CYB561D2	CTTN		CLMN	CLIP1	
CYHR1	CTTNBP2NL		CLSPN	CLIP4	
CYorf15B	CUGBP2		CLSTN2	CLOCK	
CYP11A1	CUGBP2		CLTB	CLTC	
CYP2E1	CUL2		CLUL1	CMPK	
CYP2F1	CUTL1		CMTM1	CMTM4	
CYP4A11	CXorf41		CMYA1	CNIH2	
CYP4A22	CXXC6		CNGA1	CNN1	
DAB2	CYB5A		CNGB1	CNOT2	
DBC1	CYP26B1		CNOT3	CNOT6	
DBC1	D4S234E		CNTFR	CNOT6L	
DBI	DACH1		CNTNAP3	CNOT7	
DBNDD2	DACH1		COL13A1	CNR1	
DBNDD2	DACH1		COL13A1	CNTFR	
DBNDD2	DAG1		COL16A1	CNTNAP1	
DCDC2	DAGLA		COL23A1	COBLL1	
DCLRE1A	DAPK1		COL5A1	COL10A1	
DCLRE1A	DCAMKL1		COL9A2	COL11A1	
DCN	DCBLD2		COPS4	COL12A1	
DCT	DCBLD2		CPNE2	COL19A1	
DDX31	DCBLD2		CRABP1	COL1A1	
DDX49	DCDC2		CRADD	COL1A2	
DEDD2	DCUN1D1		CRADD	COL22A1	
DEFA4	DCUN1D4		CREB3L3	COL24A1	
DEFA4	DDIT4		CREB3L4	COL4A4	
DEFB111	DDIT4		CREB3L4	COL5A1	
DEFB121	DDX17		CREB3L4	COPS2	
DEGS2	DDX3X		CREB3L4	COPS7A	
DEPDC5	DDX3X		CREM	COPS7B	
DEPDC5	DDX3Y		CRIPAK	COX5A	
DEPDC5	DEDD		CRKL	CPD	
DES	DEPDC1B		CRNKL1	CPEB2	
DES	DERL1		CRP	CPEB3	
DGAT1	DIAPH3		CRP	CPEB4	
DHX30	DIXDC1		CRTC2	CPSF2	
DHX9	DKK2		CRTC2	CREB5	
DHX9	DLC1		CSE1L	CREBBP	
DIO2	DLEU7		CSF3R	CREBL2	
DKKL1	DLG3		CSMD1	CREBZF	
DLEU1	DLG5		CSNK1G2	CRIM1	
DLGAP1	DLGAP4		CSPG5	CRKL	
DMAP1	DLL1		CST7	CRSP7	
DNAJB13	DLL1		CST9L	CRTC2	
DNAJC12	DLL4		CST9L	CRTC3	
DNASE1L2	DMRT3		CTNNAL1	CS	
DNM3	DMRT3		CTNNAL1	CSDE1	
DNPEP	DMTF1		CTNNAL1	CSMD1	
DNPEP	DMXL1		CTRL	CSNK1G1	
DNPEP	DNAJA2		CTRL	CTCF	
DNPEP	DNAJA5		CTSL2	CTDSP2	

Plasma Microvesicles Predicted Targets			PBMC Predicted Targets		
Sanger miRBase	TargetScan	Common	Sanger miRBase	TargetScan	Common
DOCK1	DNAJB12		CTSL2	CTDSPL	
DOCK6	DNAJC14		CUL4B	CTGF	
DOCK8	DNAJC16		CUL4B	CTNNBIP1	
DOK7	DND1		CUTA	CTNND2	
DPH3B	DOCK3		CUTL1	CTTNBP2NL	
DPH3B	DOCK4		CUTL1	CUGBP2	
DSG2	DOLPP1		CX04A_HUMAN	CUL2	
DTX3	DPP10		CXCL3	CUL4B	
DYNC1H1	DPYSL2		CXorf26	CUL5	
EARS2	DRAM		CXorf26	CUTL1	
ECD	DRD1		CXorf27	CX3CL1	
ECHDC2	DSEL		CYHR1	CXCL10	
EDC4	DTNA		CYorf15B	CXorf15	
EDF1	DUSP10		CYP11A1	CXorf41	
EFCAB4B	DUSP16		CYP2A7	CXorf45	
EFCAB5	DUSP3		CYP2E1	CXXC6	
EFHB	DUSP5		CYP2F1	CYB5A	
EGFL7	DYNC1I1		DAB2	CYLD	
EGLN2	DYNC1LI2		DAD1	CYorf15B	
EIF3S8	DYRK1A		DBC1	CYP26B1	
ELF3	DYRK1B		DBC1	D4S234E	
ELF3	DYRK2		DBNDD2	DAAM1	
EML3	E2F3		DBNDD2	DACH1	
EMR4	E2F7		DBNDD2	DAG1	
ENO1P	E2F7		DCPS	DAPK1	
ENOSF1	EAF1		DCT	DBN1	
ENOSF1	EDA		DDX31	DCAMKL1	
ENTPD5	EDA		DEAF1	DCBLD2	
EPHA1	EDEM3		DEDD2	DCDC2	
EPHA5	EDG1		DEFB111	DCP2	
EPHX2	EDG2		DEPDC5	DCUN1D3	
EPHX2	EGR1		DEPDC5	DCUN1D4	
EPHX2	EHD1		DEPDC5	DDEF2	
ERN2	EIF2C4		DEPDC5	DDIT4	
EXOSC10	EIF3S1		DES	DDX17	
EXOSC10	EIF3S10		DES	DDX3X	
EZH2	EIF3S10		DET1	DDX3Y	
FAM108B1	EIF4B		DGAT1	DDX6	
FAM112B	EIF4E		DHX15	DEDD	
FAM116B	EIF4E3		DHX30	DENND1A	
FAM38A	EIF4G2		DHX9	DEPDC1B	
FAM38A	EIF4G2		DHX9	DERL1	
FAM46B	EIF4G2		DIO2	DGCR8	
FAM46B	EIF5		DJBP_HUMAN	DGKG	
FAM46B	EIF5A2		DKKL1	DHDDS	
FAM54B	ELAVL1		DKKL1	DHX36	
FAM54B	ELAVL2		DLEU1	DHX40	
FAM55C	ELAVL2		DLGAP1	DIAPH3	
FAM55C	ELAVL3		DMBX1	DICER1	
FAM63A	ELL		DMBX1	DIXDC1	
FAM63A	ELL		DMKN	DKFZP564O0823	
FANCD2	ELMO2		DNAJB13	DKFZP686A01247	
FBXO2	ENAH		DNAJC1	DKK3	
FBXO2	ENSA		DNAJC12	DLC1	
FBXO44	ENTPD6		DNAJC12	DLEU7	

Plasma Microvesicles Predicted Targets			PBMC Predicted Targets		
Sanger miRBase	TargetScan	Common	Sanger miRBase	TargetScan	Common
FBXO8	EP300		DNASE1L2	DLG5	
FBXO8	EP400		DNM3	DLGAP1	
FBXW2	EPB41L3		DNPEP	DLL1	
FBXW2	EPB41L3		DNPEP	DLL4	
FBXW4	EPB41L4B		DNPEP	DLX1	
FCER1G	EPC2		DNPEP	DLX3	
FCER1G	EPHA2		DNPEP	DMRT3	
FCGR1B	EPHA7		DNPEP	DMTF1	
FCHO1	EPHA7		DOCK1	DMXL1	
FCHO2	EPS15		DOCK1	DMXL2	
FCN2	ERBB4		DOCK8	DNAJA2	
FCRL2	ERBB4		DOK7	DNAJA5	
FCRL2	ERBB4		DPH3B	DNAJB1	
FES	ERC2		DPP9	DNAJC16	
FGF8	ERC2		DSG2	DND1	
FGFBP2	ERLIN1		DTX2	DNMT3A	
FGFBP2	ERLIN2		DTX3	DOCK10	
FGFR2	ERO1LB		DTX3	DOCK3	
FGFR4	ESCO1		DUS2L	DOCK4	
FGL1	ESR1		DYNC1H1	DOLPP1	
FGL1	ESR1		DYNC1H1	DPF2	
FILIP1L	ESRRA		EARS2	DPYSL2	
FKHL18	ESRRG		ECE2	DPYSL5	
FKHL18	ETF1		ECHDC2	DRAM	
FLT3	ETNK1		EDA	DRD1	
FMO1	ETNK1		EDF1	DSEL	
FMO1	ETS1		EFCAB4B	DTNA	
FNTA	ETS2		EFCAB5	DUSP10	
FOXH1	EXOC3L2		EFHB	DUSP3	
FOXH1	EXOC5		EGLN2	DUSP5	
FREM2	EYA1		EIF3S8	DYNC1I1	
FSD1	EZH1		EIF4A1	DYNC1LI2	
FSHR	EZH1		EIF4A1	DYRK1A	
FUK	EZH2		ELOVL3	DYRK1B	
FUSIP1	F3		EMR4	E2F3	
GABARAPL2	FA2H		EMX1	E2F7	
GABARAPL2	FAM107B		ENOSF1	E2F8	
GAL3ST3	FAM116A		ENOSF1	EAF1	
GALK1	FAM116A		ENPP5	EBF2	
GALNTL4	FAM122A		ENSA	EBF3	
GART	FAM123B		ENTPD5	EDA	
GART	FAM13A1		EPHA1	EDARADD	
GBA2	FAM44B		EPHA5	EDEM3	
GBA2	FAM45A		EPHA5	EDG1	
GBX2	FAM46A		EPHX2	EDG2	
GDA	FAM46C		EPHX2	EEA1	
GDF2	FAM49B		EPHX2	EFNA1	
GDF5	FAM54B		EPHX2	EFNB1	
GDF9	FAM5C		ERICH1	EFNB2	
GFRAL	FAM60A		EXOSC10	EGLN2	
GLE1L	FAM62B		EXOSC10	EGR1	
GLMN	FAM70A		EXOSC5	EGR2	
GLS2	FAM78B		EZH2	EGR3	
GLT6D1	FAM80B		F11R	EHD1	
GLUD2	FAM81A		FADS1	EIF2C1	

Plasma Microvesicles Predicted Targets			PBMC Predicted Targets		
**Sanger miRBase**	**TargetScan**	**Common**	**Sanger miRBase**	**TargetScan**	**Common**
GLUD2	FAM91A1		FAHD1	EIF2C4	
GMPPA	FAM98A		FAM108B1	EIF3S10	
GNAL	FAT		FAM108B1	EIF4A2	
GNG11	FAT2		FAM112B	EIF4B	
GNG11	FBN2		FAM129C	EIF4E	
GOLGB1	FBXL10		FAM19A3	EIF4G2	
GOLGB1	FBXO11		FAM19A3	EIF4G3	
GOLT1A	FBXO11		FAM3B	EIF4H	
GOT2	FBXO21		FAM46B	EIF5	
GPR119	FBXO28		FAM46B	EIF5A2	
GPR142	FBXO33		FAM46B	ELAVL1	
GPR157	FBXO8		FAM55C	ELAVL2	
GPR172A	FBXW7		FAM55C	ELAVL3	
GPR172A	FBXW7		FAM57B	ELK1	
GPR34	FCHSD1		FAM59A	ELK3	
GPR63	FCHSD2		FAM63A	ELL	
GPR63	FGD1		FAM63A	ELL2	
GPR63	FGF1		FAM80A	ELMO2	
GPR63	FGF2		FAM83F	ELMOD2	
GPR63	FGF7		FAM84A	ELOVL3	
GPR89A	FGFR1		FAM84A	ELOVL5	
GPR89A	FGFR2		FAM84A	EMX2	
GPR89B	FLJ12529		FAM84A	ENAH	
GPR89B	FLJ14213		FASN	ENC1	
GPX1	FLJ14668		FASTK	ENPP5	
GPX1	FLJ20152		FAT3	ENSA	
GPX5	FLJ20186		FBXO2	EP300	
GRIA3	FLJ20309		FBXO2	EP400	
GRINA	FLJ20366		FBXO36	EPAS1	
GRK5	FLJ25476		FBXO44	EPB41L2	
GRM3	FLJ31951		FBXO8	EPB41L3	
GSDMDC1	FLJ32447		FBXO8	EPB41L4B	
GSTA2	FLJ36070		FBXO8	EPB49	
GSTT2	FLJ37357		FBXW2	EPC2	
GSTT2_HUMAN	FLJ39502		FBXW2	EPHA1	
GTF2B	FLJ40142		FBXW4	EPHA2	
GTF2B	FLJ45187		FCER1G	EPHA7	
GTPBP8	FLNA		FCER1G	EPHB2	
GTPBP8	FLT1		FCGR1B	EPHB3	
GTPBP8	FMR1		FCN2	EPN2	
GTPBP8	FNBP1L		FCN2	EPS15	
GZMK	FNDC3A		FCRL2	ERBB4	
H2AFX	FNIP1		FCRL2	ERC2	
HACL1	FNIP1		FDXR	EREG	
HAX1	FOS		FES	ERLIN1	
HBA2	FOSL1		FFAR3	ERLIN2	
HBE1	FOXN2		FGF8	ERO1LB	
HBE1	FOXO3A		FGFR2	ESCO1	
HCG9	FOXP1		FGFR4	ESR1	
HD	FOXP1		FGL1	ESRRA	
HDAC6	FREM2		FGL1	ESRRG	
HDAC8	FREQ		FKBP15	ETF1	
HDAC8	FRMPD1		FKBP8	ETNK1	
HDGF	FRS2		FKHL18	ETV1	
HDGF	FRYL		FLT3	ETV5	

Plasma Microvesicles Predicted Targets			PBMC Predicted Targets		
Sanger miRBase	TargetScan	Common	Sanger miRBase	TargetScan	Common
HEATR5B	FSD1		FNTA	EVI5L	
HECTD3	FUBP1		FOXD4L1	EXOC3L2	
HELZ	FUBP3		FOXH1	EXOC5	
HEPH	FURIN		FOXH1	EYA1	
HEPH	FUT9		FOXQ1	EZH1	
HGS	FUT9		FSD1	EZH2	
HHLA3	FVT1		FSHR	F3	
HIST1H2AM	FZD10		FUBP3	FA2H	
HIST2H2BA	FZD5		FUK	FAM104A	
HLA-C	FZD5		FUSIP1	FAM114A1	
HLA-DMA	G0S2		FX4L4_HUMAN	FAM116A	
HLA-DOA	G3BP2		FZD9	FAM120A	
HLA-G	GABRA1		GAA	FAM122A	
HLA-G	GABRA1		GABARAPL2	FAM123B	
HLA-J	GABRA1		GAK	FAM13A1	
HMBS	GABRA4		GAK	FAM38A	
HMGCL	GAD1		GALK1	FAM43A	
HNRPC	GAL3ST3		GALNTL4	FAM44B	
HNRPUL2	GALNT10		GALR2	FAM45A	
HOMER3	GALNT7		GART	FAM46A	
HOOK2	GAN		GART	FAM46B	
HOXA5	GAP43		GBX2	FAM46C	
HOXD10	GAPVD1		GDF11	FAM49B	
HOXD4	GARNL1		GDF2	FAM54B	
HOXD4	GBA2		GDF5	FAM5C	
HP	GGA3		GDPD5	FAM60A	
HPSE2	GHR		GEMIN8	FAM70A	
HR	GLUD1		GEMIN8	FAM73B	
HRH2	GLUD2		GFOD2	FAM80A	
HRK	GMFB		GFOD2	FAM80B	
HS6ST3	GNAI2		GFRAL	FAM81A	
HSD11B1	GNAI3		GJA7	FAM83D	
HSD11B1	GNPNAT1		GLE1L	FAM84A	
HSD17B6	GOLGA1		GLRA1	FAM91A1	
HSPA1A	GORASP2		GLS2	FAM98A	
HSPA8	GPATCH8		GLUD2	FANCA	
HSPA8	GPC4		GLUD2	FASTK	
HTF9C_HUMAN	GPM6A		GMPPB	FAT	
HTR3C	GPR63		GNA11	FBXL10	
HTR4	GRAMD3		GNB3	FBXL11	
HTRA1	GRB10		GNG11	FBXO10	
HTRA2	GRHL3		GNG11	FBXO11	
HUS1	GRIA3		GOLGB1	FBXO21	
HYAL1	GRIA4		GOLT1A	FBXO28	
IBRDC3	GRM7		GOT2	FBXO33	
IDH2	GRSF1		GPHA2	FBXO8	
IDH3G	GSK3B		GPR128	FBXW11	
IDH3G	GTDC1		GPR137	FBXW7	
IDUA	GTF3C2		GPR142	FCHSD1	
IDUA	H2AFX		GPR157	FCHSD2	
IFIH1	H3F3A		GPR161	FEM1C	
IFNA1	HAO1		GPR161	FGD1	
IFNG	HAS2		GPR172A	FGF1	
IFT20	HAS3		GPR27	FGF2	
IFT20	HAS3		GPR34	FGF7	

Plasma Microvesicles Predicted Targets			PBMC Predicted Targets		
Sanger miRBase	TargetScan	Common	Sanger miRBase	TargetScan	Common
IFT74	HD		GPR63	FGFR1	
IGFBPL1	HECTD1		GPR63	FGFR2	
IGKV3-20	HECTD2		GPR63	FJX1	
IGLL1	HELZ		GPX1	FLJ11783	
IHPK2	HELZ		GPX1	FLJ12529	
IK	HELZ		GPX5	FLJ14213	
IL13RA1	HHEX		GRHPR	FLJ14668	
IL1RL2	HIAT1		GRHPR	FLJ20152	
IL20	HIC2		GRIA3	FLJ20160	
IL20	HIGD1A		GRIA3	FLJ20186	
IL20RA	HIP1R		GRK4	FLJ20309	
IL29	HIPK1		GRK5	FLJ20366	
IMPG1	HIPK1		GRK5	FLJ20489	
INE1	HIRA		GRM3	FLJ25476	
INPP5B	HIVEP2		GRM3	FLJ31818	
INPP5B	HLA-DOB		GSG1L	FLJ31951	
INPP5B	HLF		GSTA2	FLJ32447	
INPP5B	HMBOX1		GSTA2	FLJ32679	
INPP5B	HMGA1		GSTT2	FLJ36070	
INPP5B	HMGA1		GSTT2_HUMAN	FLJ36874	
INTS8	HMGA2		GTF2B	FLJ37357	
INTU	HMGA2		GTPBP8	FLJ37464	
IRGQ	HMGCS1		GTPBP8	FLJ39502	
IRX4	HNRPA3		GTPBP8	FLJ40142	
IRX4	HNRPD		GTPBP8	FLJ45187	
ISOC2	HNRPUL1		GZMK	FLJ45557	
ITGB1BP3	HOOK1		GZMK	FLNA	
ITIH1	HOXA10		HACL1	FLNC	
IVD	HOXA3		HACL1	FMN2	
JAKMIP1	HOXA5		HAPLN2	FMR1	
K0401_HUMAN	HOXA5		HARS	FNBP1L	
KARS	HOXA9		HAX1	FNDC3A	
KATNB1	HOXC10		HBE1	FNDC3B	
KATNB1	HOXC11		HBE1	FNDC5	
KBTBD2	HOXC4		HCG9	FNDC8	
KCNF1	HOXC8		HCN2	FNIP1	
KCNF1	HOXD13		HD	FOSL1	
KCNH3	HOXD8		HDAC6	FOXF2	
KCNIP3	HPGD		HDAC6	FOXO3A	
KCNQ1	HRB		HDAC8	FOXP1	
KCNQ4	HSP90B1		HDAC8	FREQ	
KIAA0241	HSPA4L		HDGF	FRMPD1	
KIAA0241	HSPG2		HDGF	FRY	
KIAA0241	HSPG2		HEATR3	FRYL	
KIAA0652	HTR2A		HECTD3	FSD1	
KIAA1245	HTR4		HELZ	FSD1L	
KIAA1245	HTRA3		HELZ	FUBP1	
KIAA1245	HUWE1		HEPH	FUBP3	
KIAA1245	IBRDC1		HEPH	FUK	
KIAA1602	IBRDC2		HERC1	FURIN	
KIAA1618	IGF1R		HIPK3	FUT9	
KIAA1618	IGF1R		HIPK3	FVT1	
KIAA1731	IGF2BP2		HIST1H2AM	FZD10	
KIAA1822L	IGF2R		HLA-DMA	FZD4	
KIAA1881	IHH		HLA-DOA	FZD5	

Plasma Microvesicles Predicted Targets			PBMC Predicted Targets		
Sanger miRBase	TargetScan	Common	Sanger miRBase	TargetScan	Common
KIF20A	IL1R1		HMBS	FZD8	
KIF21A	IL1RAPL1		HMGCL	G0S2	
KIF27	IL6		HNRPC	G3BP2	
KIF27	INA		HNRPUL2	GABRA1	
KIF7	ING5		HOMER3	GABRA4	
KIFC3	INHBB		HOOK2	GABRB2	
KIR2DL1	INPP5B		HOXA5	GABRB3	
KIR2DL1	INSIG1		HOXD10	GABRG2	
KIR2DL1	INSIG1		HOXD4	GAD1	
KIR2DL1	INSR		HPSE2	GALNT10	
KIR2DL4	INTU		HR	GALNT7	
KIR3DL1	IPO11		HRASLS	GAN	
KLHDC8B	IPPK		HRH2	GAP43	
KLHL5	IPPK		HRH4	GAPVD1	
KLKB1	IRAK1		HRK	GATA4	
KLRC3	IRF2		HSCB	GATAD2A	
KRT33B	IRX5		HSD17B10	GDA	
KRT34	ISOC1		HSD17B6	GDI1	
KRT73	ITGA2		HSD17B7	GGA3	
KRT81	ITGA5		HSPA1A	GHR	
KRTAP9-9	ITGA6		HSPA1A	GJA1	
KYNU	ITGB8		HSPA8	GLCCI1	
LAMA4	ITPR1		HTF9C_HUMAN	GLDC	
LAMA5	ITPR1		HTF9C_HUMAN	GLIS2	
LAMA5	JAG1		HTF9C_HUMAN	GLIS3	
LAMB1	JAKMIP2		HTR4	GLP1R	
LAMB3	JAZF1		HTRA2	GLRA1	
LAMB3	JMJD2A		HUS1	GLUD1	
LAMB3	JUB		IBRDC3	GLUD2	
LAMB3	KBTBD2		ICAM4	GMFB	
LAMB3	KBTBD4		IDH3G	GNAI1	
LAMB3	KBTBD8		IDH3G	GNAI3	
LATS1	KCNAB1		IFI16	GNPNAT1	
LCE3D	KCNH7		IFI16	GOLGA	
LDHAL6B	KCNJ2		IFI16	GOLGA1	
LDHB	KCNJ2		IFIH1	GOLGA6	
LELP1	KCNJ2		IFNA1	GOLGA8A	
LENG8	KCNK1		IFT20	GOLGA8B	
LENG8	KCNK2		IFT20	GOLGA8E	
LHCGR	KCNN4		IFT74	GOLGA8G	
LIPE	KCNQ4		IGBP1	GORASP2	
LMO7	KCTD1		IGFBPL1	GPATCH8	
LMX1B	KCTD18		IGKV1-12	GPIAP1	
LOC642623	KCTD21		IGKV1-16	GPM6A	
LOC652153	KCTD8		IGKV1-17	GPR137B	
LOC652438	KHDRBS2		IGKV1-6	GPR63	
LOC727940	KIAA0152		IGKV1-8	GRAMD3	
LOC728350	KIAA0157		IGKV1D-12	GRB10	
LOC728932	KIAA0240		IGKV1D-13	GRHL2	
LOC729368	KIAA0241		IGKV1D-16	GRHL3	
LOC730436	KIAA0241		IGKV1D-17	GRIA3	
LOC730803	KIAA0355		IGKV1D-8	GRIA4	
LOC732382	KIAA0368		IGLL1	GRIN1	
LRFN2	KIAA0467		IGSF21	GRIN2A	
LRFN4	KIAA0528		IHPK2	GRK6	

Plasma Microvesicles Predicted Targets			PBMC Predicted Targets		
Sanger miRBase	TargetScan	Common	Sanger miRBase	TargetScan	Common
LRP10	KIAA0664		IK	GRM7	
LRRC46	KIAA0672		IL13RA1	GRSF1	
LRRC50	KIAA0828		IL1RL2	GSC	
LRRC8B	KIAA0831		IL20	GSK3B	
LRRC8D	KIAA1033		IL20	GTDC1	
LRRK1	KIAA1267		IL20	GTF2H1	
LSM10	KIAA1305		IL29	GTF3C2	
LSM10	KIAA1333		ILK	GULP1	
LSM14B	KIAA1370		IMPDH1	H1F0	
LSM2	KIAA1411		IMPDH2	HAO1	
LSM2	KIAA1468		IMPG1	HAS2	
LSM2	KIAA1468		INE1	HAS3	
LTB	KIAA1539		INOC1	HBP1	
LTB4R	KIAA1546		INPP5B	HD	
LTBP3	KIAA1598		INPP5B	HDAC4	
LTBP3	KIAA1787		INPP5B	HDAC7A	
LY6G5B	KIAA2018		INPP5B	HDGF	
LY6G6C	KIF1B		INPP5B	HDLBP	
LY6H	KIF21A		INPP5B	HECTD1	
LYPLA2	KIF21B		INTS8	HECTD3	
LYZL4	KIF23		INTU	HECW2	
MAD2L2	KIF3B		IPO13	HELZ	
MAD2L2	KIF5A		IQWD1	HHEX	
MAD2L2	KIF5B		IQWD1	HIAT1	
MAD2L2	KIF5C		IQWD1	HIC2	
MAD2L2	KIT		IRGQ	HIF1A	
MAEL	KLC1		IRGQ	HIG2	
MAEL	KLC4		IRX4	HIGD1A	
MAGEB2	KLF10		IRX4	HIPK1	
MAGED1	KLF12		ITIH1	HIPK2	
MAGED1	KLF7		IVD	HIPK3	
MAGED1	KLHDC5		IVD	HIRA	
MAGEE1	KLHL1		JAKMIP1	HIVEP2	
MAN2A1	KLHL18		JAKMIP1	HLA-DOB	
MAP1LC3A	KLHL18		JAKMIP1	HLF	
MAP1LC3A	KLHL2		JTB	HMBOX1	
MAP3K10	KLHL28		JTB	HMGA1	
MAP3K11	KLHL3		K0401_HUMAN	HMGA2	
MBOAT1	KPNA2		KARS	HMGCS1	
MCM3AP	KPNA2		KATNB1	HNRPA1	
MCOLN3	KPNA3		KATNB1	HNRPC	
MDH1B	KPNA3		KBTBD2	HNRPD	
MDK	KPNA3		KBTBD5	HNRPF	
MDN1	KPNA4		KBTBD5	HNRPU	
MEA1	KRTAP11-1		KCNA2	HNRPUL1	
MED11	KRTAP4-4		KCNH3	HOOK1	
MEIG1	KSR1		KCNIP3	HOXA10	
MESDC1	Kua		KCNQ1	HOXA3	
MIA	Kua-UEV		KCNQ4	HOXA5	
MIA	LAMC1		KCTD4	HOXA9	
MIA	LARP1		KCTD4	HOXC11	
MIA	LARP4		KIAA0241	HOXC4	
MLLT10	LARP5		KIAA0241	HOXC8	
MLLT10	LAT		KIAA1033	HOXD13	
MLLT7	LATS2		KIAA1245	HOXD8	

Plasma Microvesicles Predicted Targets			PBMC Predicted Targets		
Sanger miRBase	TargetScan	Common	Sanger miRBase	TargetScan	Common
MLN	LBR		KIAA1245	HPGD	
MMP21	LEF1		KIAA1245	HSP90B1	
MMP23A	LELP1		KIAA1245	HSPA4L	
MOGAT3	LENG4		KIAA1618	HSPG2	
MORG1_HUMAN	LGR4		KIAA1822L	HTR2A	
MORN2	LHFPL2		KIF21A	HTR2C	
MORN2	LHFPL2		KIF27	HTR4	
MOSPD3	LHX9		KIF27	HUWE1	
MPDU1	LIF		KIF7	IBRDC1	
MPDU1	LIFR		KIF9	IBRDC2	
MPI	LIMD2		KIR2DL1	ICK	
MPST	LIN54		KIR2DL1	ID2	
MPZL1	LINGO1		KIR2DL1	ID4	
MR1	LITAF		KIR2DL1	IGF1	
MRAP	LMAN2L		KIR2DL4	IGF1R	
MRO	LMBR1L		KIR3DL1	IGF2R	
MRPL1	LMO2		KLHDC2	IGFBP3	
MRPL37	LMTK2		KLHDC8B	IGSF3	
MRPL43	LOC153222		KLHL22	IHH	
MRPL48	LOC153222		KLKB1	IL17RD	
MRPS30	LOC203547		KLRC3	IL1RAPL1	
MRPS7	LOC203547		KRT15	IL6	
MSRB2	LOC283514		KRT23	IMPDH1	
MT1JP	LOC285382		KRT33B	ING4	
MT1JP	LOC345222		KRT34	INHBB	
MT1M	LOC400258		KRT73	INOC1	
MTMR14	LOC57228		KRTAP9-9	INPP5A	
MTMR14	LOC91461		KY	INPP5B	
MTTP	LPHN1		LAMA5	INSR	
MTX2	LPHN2		LAMA5	INTU	
MUC20	LPXN		LAMB1	IPO11	
MUC20	LRFN2		LAMB3	IPPK	
MUSTN1	LRFN2		LAMB3	IQWD1	
MX1	LRIG1		LAMB3	IRAK1	
MYL2	LRIG2		LAMB3	IRAK2	
MYO18A	LRP1B		LAMB3	ISOC1	
MYO3A	LRP2		LAMB3	ITCH	
MYO7B	LRP6		LARP7	ITFG3	
MYOD1	LRRC15		LATS1	ITGA2	
MYPN	LRRC16		LCN6	ITGA5	
MYST1	LRRFIP2		LDHB	ITGA6	
MYST1	LRRN1		LELP1	ITGB1	
MYST1	LRRN3		LENG8	ITGB8	
NAPRT1	LRRN3		LENG8	ITPKB	
NAT14	LRRTM4		LEPREL2	ITPR1	
NBR2	LRTM2		LIG1	ITSN1	
NCBP1	LSM11		LIPE	IVNS1ABP	
NCR1	LSM12		LLGL2	JAG1	
NCR3	LTBP1		LLGL2	JAKMIP1	
NCR3	LUZP1		LMO6	JAKMIP2	
NDUFB8	LYPLA3		LMTK2	JAZF1	
NDUFS3	LYRM1		LMX1B	JMJD2A	
NECAP2	LYRM5		LOC642623	JMJD3	
NEIL1	M6PR		LOC642623	KBTBD2	
NEK10	MAB21L1		LOC647591	KBTBD4	

Plasma Microvesicles Predicted Targets			PBMC Predicted Targets		
Sanger miRBase	TargetScan	Common	Sanger miRBase	TargetScan	Common
NELFB_HUMAN	MACF1		LOC652153	KBTBD8	
NENF	MAFB		LOC652438	KCNA4	
NFE2	MAG1		LOC727940	KCNAB1	
NFKBIL1	MAGI1		LOC727940	KCNH7	
NGRN	MAN2A1		LOC729368	KCNIP1	
NGRN	MAP1B		LOC729368	KCNJ2	
NIM1_HUMAN	MAP1B		LOC730436	KCNK1	
NLRP14	MAP2		LOC730436	KCNK2	
NM_001013739.1	MAP2		LOC732382	KCNN3	
NM_003585.2	MAP2K1		LRFN4	KCNN4	
NM_138781	MAP3K11		LRP2	KCNQ4	
NM_173565.2	MAP3K12		LRP2BP	KCNQ5	
NM_207480	MAP3K2		LRP5	KCNS2	
NME1	MAP3K3		LRRC27	KCTD1	
NOL4	MAP3K7IP3		LRRC37A3	KCTD15	
NOLA1	MAP3K7IP3		LRRC46	KCTD18	
NOVA2	MAP7		LRRC61	KCTD8	
NOX4	MAPK10		LRRC8B	KIAA0141	
NP_001001694.1	MAPK6		LRRC8D	KIAA0152	
NP_001005303.1	MAPK7		LRRK1	KIAA0240	
NP_001007538.1	MAPKAPK2		LSM1	KIAA0241	
NP_001008396.1	MAPRE1		LSM10	KIAA0280	
NP_001017927.1	MAPRE3		LSM14B	KIAA0355	
NP_001020528.1	MARCKSL1		LSM14B	KIAA0367	
NP_001025037.1	MARK1		LSM2	KIAA0423	
NP_001032755.1	MARK1		LSM2	KIAA0528	
NP_001034637.1	MARK4		LSM2	KIAA0664	
NP_001072995.1	MAT2A		LTB	KIAA0828	
NP_001072996.1	MAT2A		LTBP3	KIAA0831	
NP_001072997.2	MATN3		LTBP3	KIAA0907	
NP_001073912.1	MATR3		LTV1	KIAA1033	
NP_001073912.1	MBD6		LY6G5B	KIAA1128	
NP_001073931.1	MBOAT1		LYPLA3	KIAA1212	
NP_001073999.1	MBP		LYZL4	KIAA1217	
NP_001073999.1	MCHR1		MAD2L2	KIAA1219	
NP_056002.1	MCL1		MAD2L2	KIAA1305	
NP_056263.1	MEF2C		MAEL	KIAA1333	
NP_056263.1	MEGF9		MAEL	KIAA1411	
NP_056519.1	MEN1		MAGEB2	KIAA1468	
NP_060404.3	MESDC1		MAGED1	KIAA1539	
NP_065789.1	MFHAS1		MAGED1	KIAA1553	
NP_077297.2	MGAT4A		MAGED1	KIAA1598	
NP_077297.2	MGAT4A		MAGEE1	KIAA1787	
NP_115767.1	MGC40405		MAGEE1	KIF1B	
NP_219485.1	MIB1		MAL	KIF1C	
NP_612480.1	MIB1		MAN2A1	KIF21A	
NP_631913.2	MIDN		MAP3K11	KIF21B	
NP_665806.1	MIDN		MAP3K11	KIF23	
NP_694957.2	MIER3		MAPK13	KIF3A	
NP_699199.2	MIER3		MAST2	KIF4A	
NP_775836.2	MIPOL1		MBD4	KIF5A	
NP_775916.1	MITF		MBD6	KIF5B	
NP_840059.1	MKNK2		MCF2L	KIF5C	
NP_840059.1	MLL		MCM3AP	KIT	
NP_847884.1	MLL		MDH1B	KLC2	

Plasma Microvesicles Predicted Targets			PBMC Predicted Targets		
Sanger miRBase	TargetScan	Common	Sanger miRBase	TargetScan	Common
NP_872380.1	MLL3		MDK	KLC4	
NP_872380.1	MLL3		MEA1	KLF10	
NP_945352.1	MLLT6		MED11	KLF12	
NP_997719.2	MMD		MED4	KLF13	
NPBWR2	MMP14		MEIG1	KLF4	
NPC2	MMP16		MFN1	KLF7	
NPDC1	MMP16		MFN1	KLHDC5	
NPEPL1	MMP16		MFSD5	KLHL18	
NPR2	MNT		MIA	KLHL2	
NR_002162.1	MNT		MIA	KLHL20	
NR0B1	MOBK1B		MIER1	KLHL28	
NR1H2	MOBKL2B		MIZF	KLHL3	
NR1I3	MOV10		MLH3	KPNA1	
NR4A3	MPPE1		MLLT10	KPNA2	
NSL1	MPZ		MLLT10	KPNA3	
NSMCE4A	MREG		MLLT4	KPNA4	
NT5DC4	MRPL43		MLN	KRAS	
NUDT22	MSL2L1		MLN	KRCC1	
NUPR1_HUMAN	MTDH		MLRM_HUMAN	KRTAP11-1	
NXF1	MTF2		MMP21	KRTAP4-4	
O75264_HUMAN	MTHFR		MORG1_HUMAN	Kua	
ODF3	MTMR14		MORN2	Kua-UEV	
ODF3	MTMR4		MORN2	L3MBTL3	
ODF4	MTPN		MOSC1	LAMC1	
ODF4	MTPN		MOV10L1	LAMP2	
ODZ3	MTX2		MPDU1	LARP1	
OFCC1	MUM1L1		MPDU1	LARP4	
OGFR	MXI1		MPST	LARP5	
OLFM1	MXI1		MR1	LAT	
OMG	MYB		MRPL1	LATS2	
ONECUT1	MYBL1		MRPL12	LBH	
OPRD1	MYBL1		MRPL12	LBR	
OPRL1	MYBL1		MRPL14	LEF1	
OR1E2	MYBL1		MRPL37	LELP1	
OR56B1	MYCBP		MRPL43	LEMD3	
OR56B1	MYCBP2		MRPL43	LENG9	
OR5J2	MYLIP		MRPL43	LGR4	
OR6C6	MYLK		MRPL48	LHFPL4	
OR8U1	MYO10		MRPL48	LIF	
OVCH1	MYO5B		MRPS30	LIMD2	
PADI4	MYOZ3		MT1B	LIN28	
PARP9	MYST3		MTA2	LIN54	
PAX9	MYST4		MTTP	LIN9	
PCBP3	MYT1		MTX2	LINGO1	
PCDH21	MYT1L		MUSTN1	LITAF	
PCNA	N4BP1		MX1	LMAN2L	
PDCD11	NAB1		MYBL2	LMO2	
PDE2A	NAGPA		MYBPC3	LMO4	
PDE6A	NAP1L5		MYO18A	LNPEP	
PDHX	NAP1L5		MYO3A	LOC153222	
PDLIM7	NARG1		MYO7B	LOC162073	
PDLIM7	NARG1		MYPN	LOC203547	
PEMT	NAT13		MYST4	LOC220594	
PEPP2_HUMAN	NAV1		NACA	LOC285382	
PER1	NAV1		NACA3P	LOC345222	

Plasma Microvesicles Predicted Targets			PBMC Predicted Targets		
Sanger miRBase	TargetScan	Common	Sanger miRBase	TargetScan	Common
PES1	NAV2		NAP1L2	LOC401720	
PES1	NCOA1		NAT14	LOC440742	
PEX16	NDFIP2		NCBP1	LOC91461	
PFKFB3	NDFIP2		NCBP2	LONRF1	
PFKFB3	NDST1		NCR1	LPHN1	
PFN4	NEBL		NCR3	LPHN2	
PFN4	NEDD9		NCR3	LPP	
PGBD1	NEFM		NDUFA10	LPXN	
PGLS	NEK4		NECAP2	LRCH2	
PGLYRP1	NEK6		NEIL1	LRIG1	
PHF1	NET1		NEK10	LRIG2	
PHF20L1	NEUROD1		NEK11	LRP1B	
PHF5A	NFATC3		NEK8	LRP2	
PICK1	NFATC4		NELF	LRP6	
PIGB	NFE2L1		NELFB_HUMAN	LRRC15	
PIGT	NFIA		NELFB_HUMAN	LRRC16	
PIGT	NFIB		NENF	LRRC19	
PIGT	NFXL1		NFE2	LRRC55	
PIK3C2B	NHS		NFE2	LRRC8A	
PIK3CD	NIPBL		NFKBIL1	LRRFIP2	
PIK3CD	NKTR		NGRN	LRRK1	
PIM3	NLK		NGRN	LRRN3	
PIM3	NLK		NIM1_HUMAN	LRRTM4	
PIR	NLK		NKD2	LRTM2	
PIWIL4	NLRP3		NLRP14	LSM11	
PKM2	NOL4		NM_006651.3	LSM12	
PLA2G12B	NOPE		NM_138781	LTBP1	
PLAUR	NOVA1		NM_173565.2	LUZP1	
PLCB2	NOVA1		NM_173565.2	LY6E	
PLCB2	NPAT		NM_207362.2	LYCAT	
PLCD1	NRCAM		NM_207477	LYPLA2	
PLCD1	NRIP1		NM_207480	LYPLA3	
PLCL2	NRK		NME1	LYRM5	
PLCXD2	NRP2		NME1	LYST	
PLCZ1	NRP2		NME7	M6PR	
PLD3	NSMCE4A		NOL4	MAB21L1	
PLEKHA8	NTF3		NOLA1	MAB21L2	
PLEKHA8	NTN4		NOLA1	MACF1	
PLEKHC1	NUMB		NOS2A	MAF	
PLEKHO1	NUP153		NOS2A	MAFB	
PLEKHO1	NUP50		NOVA2	MAGI2	
PLK2	NUP50		NOX4	MAMDC1	
PLK2	NUS1		NOX4	MAN2A1	
PLSCR4	NUTF2		NP_001001786.1	MAP1A	
PLUNC	NXPH1		NP_001005303.1	MAP1B	
PLUNC	OAF		NP_001008396.1	MAP2	
PLUNC	OBFC2A		NP_001013739.1	MAP2K1	
PLXNA4A	OCRL		NP_001013739.1	MAP2K3	
PLXNB3	ODZ2		NP_001017927.1	MAP2K4	
PML	OGT		NP_001020528.1	MAP3K11	
PML	OGT		NP_001025037.1	MAP3K12	
PMPCB	OGT		NP_001025056.1	MAP3K14	
PMPCB	OMG		NP_001028721.1	MAP3K2	
PMVK	ONECUT1		NP_001072996.1	MAP3K3	
PODN	ONECUT2		NP_001072996.1	MAP3K4	

Plasma Microvesicles Predicted Targets			PBMC Predicted Targets		
Sanger miRBase	TargetScan	Common	Sanger miRBase	TargetScan	Common
PODN	ONECUT2		NP_001073912.1	MAP3K7IP2	
POLE4	OSBPL11		NP_001073912.1	MAP3K7IP3	
POLR1C	OSBPL2		NP_001073931.1	MAP3K8	
POLR2A	OTUD4		NP_001073948.1	MAP4K2	
POLR2H	OTUD4		NP_001073999.1	MAP7	
POLR2J	OTUD4		NP_056002.1	MAP9	
PON1	OTUD5		NP_056263.1	MAPK14	
POR	OXSR1		NP_056263.1	MAPK3	
POR	PAFAH1B1		NP_056263.1	MAPK6	
POU5F1P1	PAIP1		NP_056496.1	MAPKAPK2	
POU5F1P1	PAIP2		NP_056519.1	MAPRE1	
PPA1	PAK1		NP_056996.2	MAPRE3	
PPAP2A	PAK2		NP_060404.3	MARK1	
PPBP	PAK4		NP_061959.2	MARK2	
PPCS	PAK7		NP_065789.1	MAT2A	
PPIH	PALMD		NP_065930.2	MATR3	
PPP1R11	PAM		NP_077297.2	MBD6	
PPP1R12B	PAN3		NP_077297.2	MBNL1	
PPP1R3D	PAPD4		NP_077297.2	MBNL2	
PPP2R3C	PAPD5		NP_077297.2	MBP	
PPP2R5C	PAPPA		NP_079466.2	MCL1	
PPP4R1L	PAPPA		NP_219485.1	MDFIC	
PRAME	PAWR		NP_612362.2	MECP2	
PRAMEF5	PBEF1		NP_612448.1	MED12L	
PRKCD	PBX3		NP_612480.1	MEF2C	
PRKCD	PBX3		NP_631913.2	MEF2D	
PRMT2	PCBP4		NP_631913.2	MEMO1	
PRPS1	PCDH10		NP_659416.1	MFHAS1	
PRR11	PCDH17		NP_659482.3	MGA	
PRR6	PCDH18		NP_660305.2	MGAT4A	
PRR6	PCDH19		NP_689963.2	MGC40405	
PRRX2	PCDH9		NP_689973.2	MGC61598	
PRSS36	PCDH9		NP_694957.2	MGEA5	
PRSS7	PCDHA1		NP_694957.2	MIB1	
PRTN3	PCDHA1		NP_699199.2	MID1IP1	
PSG2	PCDHA10		NP_775836.2	MIER1	
PSMB7	PCDHA10		NP_775836.2	MIER3	
PSMB8	PCDHA12		NP_817124.1	MINK1	
PSMB8	PCDHA12		NP_847884.1	MIPOL1	
PSMC6	PCDHA13		NP_872380.1	MITF	
PSMD7	PCDHA13		NP_872380.1	MKNK2	
PTAFR	PCDHA2		NP_940961.1	MLL	
PTGDS	PCDHA2		NP_997719.2	MLL2	
PTH	PCDHA3		NP_997719.2	MLL3	
PTS	PCDHA3		NPBWR2	MLLT6	
PUNC	PCDHA4		NPC2	MLLT7	
PWP2	PCDHA4		NPDC1	MMD	
PXDNL	PCDHA5		NPEPL1	MMP14	
PXMP4	PCDHA5		NPFFR2	MMP16	
PXMP4	PCDHA6		NPR2	MN1	
Q2EN02_HUMAN	PCDHA6		NR_002162.1	MNT	
Q4G172_HUMAN	PCDHA7		NR_002593.1	MOBK1B	
Q5JQE8_HUMAN	PCDHA7		NR_002827.1	MOBKL2B	
Q5T0Z8_HUMAN	PCDHA8		NR_003034.1	MOBKL2C	
Q6UVX0_HUMAN	PCDHA8		NR0B1	MON2	

Plasma Microvesicles Predicted Targets			PBMC Predicted Targets		
Sanger miRBase	TargetScan	Common	Sanger miRBase	TargetScan	Common
Q6UXS0_HUMAN	PCDHAC1		NR1H2	MOV10	
Q6UXU7_HUMAN	PCDHAC1		NR4A3	MPHOSPH9	
Q6UY26_HUMAN	PCDHAC2		NRL	MPPE1	
Q6VEP3_HUMAN	PCDHAC2		NSL1	MPPED2	
Q6ZP22_HUMAN	PCGF5		NSUN5	MPZ	
Q6ZRV4_HUMAN	PCK1		NT5DC4	MRC2	
Q6ZTH0_HUMAN	PCMT1		NUDT16P	MREG	
Q6ZUU7_HUMAN	PCNX		NUDT17	M-RIP	
Q6ZV34_HUMAN	PCNX		NUDT22	MRPS27	
Q6ZVD1_HUMAN	PCTK2		NUP133	MTCH2	
Q6ZVY1_HUMAN	PCTK2		NXF1	MTDH	
Q76B61_HUMAN	PCTK2		NXF3	MTF1	
Q7Z2R7_HUMAN	PDCD10		OCEL1	MTF2	
Q86TU9_HUMAN	PDCD10		ODF3	MTMR10	
Q86U89_HUMAN	PDCD4		ODF3	MTMR4	
Q8IUR1_HUMAN	PDE11A		ODF4	MTPN	
Q8IUS2_HUMAN	PDE4D		ODF4	MTSS1	
Q8IVR1_HUMAN	PDE4D		OFCC1	MTUS1	
Q8N0W1_HUMAN	PDGFA		OGFR	MTX2	
Q8N1R0_HUMAN	PDGFA		OLFM1	MUM1L1	
Q8N402_HUMAN	PDGFRA		OMG	MXD4	
Q8N6V7_HUMAN	PDGFRA		ONECUT1	MXI1	
Q8TEB0_HUMAN	PDHX		OPRD1	MYADM	
Q8WWB0_HUMAN	PDIA6		OPRL1	MYB	
Q96CK5_HUMAN	PDIK1L		OR13J1	MYBL1	
Q96FF7_HUMAN	PDK4		OR1E2	MYCBP	
Q96FU4_HUMAN	PDLIM5		OR5J2	MYCBP2	
Q96HZ0_HUMAN	PDPR		OR5J2	MYCL1	
Q96IP2_HUMAN	PELI2		P2RX2	MYCN	
Q96IP2_HUMAN	PER2		P2RY11	MYLIP	
Q96M56_HUMAN	PERQ1		PAFAH2	MYLK	
Q96MA9_HUMAN	PEX13		PAK3	MYO5B	
Q96NE6_HUMAN	PEX5		PARD6A	MYO6	
Q96RW6_HUMAN	PFDN4		PARK7	MYOZ3	
Q96SQ3_HUMAN	PFKFB2		PARP9	MYST3	
Q96SQ3_HUMAN	PFKFB3		PAX9	MYT1	
Q9BR82_HUMAN	PFTK1		PBX1	MYT1L	
Q9BR82_HUMAN	PGBD5		PCBP3	N4BP1	
Q9BW98_HUMAN	PGRMC2		PCCA	NAB1	
Q9NWC8_HUMAN	PHACTR2		PCDH11Y	NAGPA	
Q9NWJ7_HUMAN	PHF19		PCDH21	NAP1L5	
Q9P181_HUMAN	PHF2		PCMT1	NAP5	
Q9UI51_HUMAN	PHF20		PCNA	NAPB	
Q9UN38_HUMAN	PHF20L1		PDCD11	NARG1	
Q9UN38_HUMAN	PHF20L1		PDE4DIP	NAT13	
R3HDML	PHF21A		PDHA1	NAT8L	
RAB3IP	PHF23		PDHX	NAV1	
RAB40A	PHF3		PDLIM7	NAV2	
RAB40A	PHF6		PDZD11	NAV3	
RABGAP1L	PHIP		PDZD11	NBEA	
RABGAP1L	PHLDB2		PEMT	NCALD	
RABGAP1L	PHLPPL		PEPP2_HUMAN	NDFIP2	
RAD54L	PHOX2B		PEPP2_HUMAN	NDST1	
RAD54L	PHTF2		PEX16	NEBL	
RAD9B	PID1		PFKFB3	NEDD8	

Plasma Microvesicles Predicted Targets			PBMC Predicted Targets		
Sanger miRBase	TargetScan	Common	Sanger miRBase	TargetScan	Common
RALGPS1	PIGS		PFKFB3	NEFM	
RAP1GAP	PIK3R1		PFN4	NEK6	
RAPH1	PIM1		PFN4	NELL2	
RARRES1	PIM1		PGBD1	NEUROD1	
RASAL2	PIM2		PGLS	NFASC	
RASGRP2	PISD		PHF1	NFATC3	
RASIP1	PITPNA		PHF1	NFATC4	
RASIP1	PITPNC1		PHF20L1	NFE2L1	
RBBP8	PKN2		PHF5A	NFIA	
RBBP8	PKNOX1		PHPT1	NFIB	
RBBP8	PLAG1		PICK1	NHS	
RBM3	PLAGL1		PIGB	NIPA1	
RBM35B	PLAGL2		PIK3CD	NIPBL	
RBM6	PLCB1		PIK3CD	NKD1	
RBMY1J	PLCD1		PIM3	NKTR	
RBP2	PLEKHA1		PIM3	NLGN1	
RBP4	PLEKHC1		PISD	NLGN2	
RBX1	PLEKHC1		PITPNM1	NLK	
REPS1	PLEKHH1		PIWIL4	NLRP3	
RFX4	PLEKHH1		PIWIL4	NME7	
RFXANK	PLEKHH1		PKM2	NMT2	
RGS22	PLK3		PKP3	NOL4	
RHBDL1	PLOD2		PKP3	NOTCH3	
RIBC1	PLOD2		PLA2G12B	NOVA1	
RIN1	PLP1		PLA2G12B	NPAS2	
RIPK3	PLP1		PLAU	NPAS3	
RIT2	PLXNA2		PLAUR	NPAS4	
RNF190	PLXNA2		PLAUR	NPEPL1	
RNF32	PMP2		PLCB3	NPTN	
RPA2	PNPLA6		PLCD1	NR2F2	
RPA4	PNRC1		PLCD1	NR3C2	
RPL21	POF1B		PLCH2	NRBF2	
RPL22L1	POGZ		PLCL2	NRCAM	
RPL34	POGZ		PLCXD2	NRF1	
RPS12	POLR3F		PLCZ1	NRIP1	
RPS15	POM121		PLD3	NRK	
RPS19BP1	POM121		PLEC1	NRP2	
RPS21	POU3F2		PLEKHA8	NRXN2	
RPS4X	PPAP2A		PLEKHC1	NRXN3	
RPS6KA6	PPARGC1A		PLEKHH3	NTN4	
RRAGA	PPM1D		PLEKHO1	NTRK3	
RRAGA	PPM1E		PLEKHO1	NUMB	
RSHL2	PPM1G		PLK2	NUP153	
RTN2	PPP1R11		PLK2	NUP50	
RTP4	PPP1R11		PLK2	NUS1	
RUFY3	PPP1R12B		PLK2	NUTF2	
S100B	PPP1R15B		PLOD3	NXN	
S100P	PPP1R15B		PLSCR4	NXPH1	
S100P	PPP2R5C		PLUNC	OAF	
SAA3P	PPP3CB		PLUNC	OBFC2A	
SALL4	PPP3CB		PLXNA4A	OCRL	
SAMD11	PPP3R1		PLXNB1	ODZ2	
SARS2	PPP3R1		PLXNB1	OGT	
SCAMP3	PPP6C		PLXNB3	OMG	
SCAMP3	PRDM1		PLXNB3	ONECUT2	

Plasma Microvesicles Predicted Targets			PBMC Predicted Targets		
Sanger miRBase	TargetScan	Common	Sanger miRBase	TargetScan	Common
SCAND1	PRDM4		PLXNC1	ORMDL2	
SCAND1	PREI3		PML	OSBPL11	
SCN2A	PRKAR2A		PML	OSBPL2	
SCN3A	PRKCD		PML	OTUB1	
SCN8A	PRKG1		PML	OTUD4	
SCOC	PRKRIP1		PMPCB	OTUD7B	
SDCCAG8	PRM1		PMPCB	OTX1	
SDF2	ProSAPiP1		PMVK	OXSR1	
SEC61A2	PRPF38A		POLE4	PA2G4	
SELENBP1	PRPF4B		POLR2A	PAFAH1B1	
SELENBP1	PRRT2		POLR2H	PAK1	
SELK_HUMAN	PSAP		PON1	PAK2	
SELO_HUMAN	PSD3		POR	PAK6	
SELV_HUMAN	PSKH1		POR	PAK7	
SEMA6D	PSMD3		POU5F1P1	PALLD	
SENP5	PSME3		POU5F1P1	PALM	
SENP8	PTBP2		POU5F1P1	PALM2	
SEP15_HUMAN	PTCH1		POU5F1P1	PALMD	
SERHL	PTCHD1		PPA1	PAM	
SERPINA3	PTEN		PPAP2A	PAN3	
SERPINA4	PTER		PPBP	PAPD4	
SERPINB4	PTGER4		PPIB	PAPD5	
SERPINI1	PTGER4		PPIE	PAPPA	
SESN3	PTGFRN		PPIH	PAWR	
SETX	PTGS2		PPM1H	PBEF1	
SFRS9	PTH		PPP1R11	PBX3	
SFT2D3	PTP4A1		PPP1R12B	PC-3	
SGCA	PTPN13		PPP1R12B	PCAF	
SGMS2	PTPN3		PPP1R3D	PCBP4	
SH2D2A	PTPN9		PPP2R3C	PCDH10	
SH2D6	PTPN9		PPP2R5C	PCDH17	
SH3GLB2	PTPRA		PPP3R1	PCDH18	
SH3GLB2	PTPRD		PPYR1	PCDH19	
SHB	PTPRD		PPYR1	PCDH9	
SIAH1	PTPRD		PRAME	PCDHA1	
SIAH1	PTPRE		PRAMEF5	PCDHA10	
SIT1	PTPRF		PRAMEF5	PCDHA12	
SIT1	PTPRF		PRAMEF6	PCDHA13	
SLC10A4	PTPRZ1		PRKCD	PCDHA2	
SLC12A5	PURA		PRMT2	PCDHA3	
SLC12A6	PURA		PRPS1	PCDHA4	
SLC12A6	PURA		PRR11	PCDHA5	
SLC13A2	PURA		PRR6	PCDHA6	
SLC16A13	PURA		PRRX2	PCDHA7	
SLC17A2	PURB		PRSS7	PCDHA8	
SLC22A15	PURB		PRTN3	PCDHAC1	
SLC22A20	PVRL1		PSG6	PCDHAC2	
SLC22A9	PVRL2		PSG9	PCGF5	
SLC25A15	QKI		PSG9	PCK1	
SLC25A15	RAB10		PSMC1	PCMT1	
SLC25A35	RAB10		PSMC1	PCNX	
SLC25A37	RAB10		PSMC6	PCTK2	
SLC25A39	RAB11FIP2		PSMC6	PDAP1	
SLC25A39	RAB1A		PSMD11	PDCD10	
SLC26A6	RAB4B		PSMD11	PDCD4	

Plasma Microvesicles Predicted Targets			PBMC Predicted Targets		
Sanger miRBase	TargetScan	Common	Sanger miRBase	TargetScan	Common
SLC26A6	RAB5C		PSMD13	PDE11A	
SLC27A2	RAB6IP1		PSMD13	PDE4D	
SLC27A2	RAB6IP1		PSMD7	PDE5A	
SLC2A13	RAB8B		PTAFR	PDE7B	
SLC5A2	RAD23B		PTCH2	PDGFA	
SLC7A10	RAD9A		PTH	PDGFRA	
SLCO3A1	RALA		PTMS	PDHX	
SLCO4A1	RALA		PTOV1	PDIA6	
SLCO4A1	RALGDS		PTPRD	PDIK1L	
SLCO4A1	RANBP10		PTS	PDK4	
SLURP1	RANBP10		PTTG1	PDLIM5	
SMARCA4	RANBP2		PXDNL	PDPK1	
SMARCA4	RANBP9		Q0VAF8_HUMAN	PDPR	
SMF_HUMAN	RAP1A		Q2EN02_HUMAN	PELI2	
SMOX	RAP1A		Q4G172_HUMAN	PER1	
SMOX	RAP1B		Q5JQE8_HUMAN	PERQ1	
SMYD2	RAP2A		Q5QHF1_HUMAN	PEX13	
SMYD2	RAP2C		Q5T400_HUMAN	PEX5	
SMYD3	RAP2C		Q6UXP8_HUMAN	PEX5L	
SNCA	RAP2C		Q6UXS0_HUMAN	PFDN4	
SNCG	RARB		Q6UXU7_HUMAN	PFKFB2	
SNCG	RARB		Q6UY26_HUMAN	PFKFB3	
SNHG5	RASA1		Q6ZNV2_HUMAN	PFN1	
SNHG5	RASA1		Q6ZP22_HUMAN	PFN2	
SNHG5	RASGEF1B		Q6ZP63_HUMAN	PFTK1	
SNRPA1	RASSF5		Q6ZR35_HUMAN	PGBD5	
SNRPC	RBBP6		Q6ZRV4_HUMAN	PGM2L1	
SNRPC	RBM16		Q6ZRV4_HUMAN	PGRMC2	
SON	RBM24		Q6ZSU1_HUMAN	PHACTR2	
SPACA4	RBM24		Q6ZSV9_HUMAN	PHC2	
SPATA2L	RBM6		Q6ZTH0_HUMAN	PHF12	
SPATA5	RBM9		Q6ZUP2_HUMAN	PHF13	
SPCS1	RBPJ		Q6ZUU7_HUMAN	PHF15	
SPHK2	RCBTB1		Q6ZUY4_HUMAN	PHF17	
SPINK7	RCN1		Q6ZV34_HUMAN	PHF19	
SPNS3	RCN2		Q6ZVN9_HUMAN	PHF20	
SPON2	RCN2		Q6ZVP1_HUMAN	PHF20L1	
SPON2	RCOR1		Q6ZVY1_HUMAN	PHF21A	
SPSB2	RECK		Q71RC1_HUMAN	PHF23	
SPSB3	REEP1		Q7Z2R7_HUMAN	PHF3	
SPTBN1	REEP3		Q86TU9_HUMAN	PHF6	
SPTBN1	REEP4		Q86U89_HUMAN	PHIP	
SRGAP2P1	RELN		Q86U89_HUMAN	PHLDA3	
SRP19	REPS2		Q8IUR1_HUMAN	PHLDB2	
SRPK3	RET		Q8IVR1_HUMAN	PHLPPL	
SRPK3	REV3L		Q8MH63_HUMAN	PHOX2B	
SRPK3	RFXDC2		Q8N0W1_HUMAN	PHTF2	
SRPRB	RFXDC2		Q8N2W8_HUMAN	PIB5PA	
SRPRB	RGS4		Q8N6F0_HUMAN	PID1	
SRPX	RHBDL1		Q8N9Z1_HUMAN	PIK3R1	
SRPX	RHOB		Q8NAM0_HUMAN	PIK3R3	
SSRP1	RHOQ		Q8ND95_HUMAN	PIK4CB	
SSU72	RICTOR		Q8TCI8_HUMAN	PIM1	
SSX6	RIMS1		Q8TEB0_HUMAN	PIP5K1B	
SSX6	RIMS3		Q8WWB0_HUMAN	PIP5K2B	

Plasma Microvesicles Predicted Targets			PBMC Predicted Targets		
Sanger miRBase	TargetScan	Common	Sanger miRBase	TargetScan	Common
STAMBPL1	RKHD2		Q96FF7_HUMAN	PISD	
STARD3NL	RKHD2		Q96IP2_HUMAN	PITPNA	
STK19	RKHD3		Q96IP2_HUMAN	PITPNC1	
STK33	RLF		Q96MA9_HUMAN	PITPNM2	
STMN1	RND3		Q96NE6_HUMAN	PITX1	
STOX2	RNF11		Q96RW6_HUMAN	PKN2	
STRBP	RNF111		Q96SQ3_HUMAN	PKNOX1	
STRBP	RNF12		Q96SQ3_HUMAN	PKP4	
STRBP	RNF125		Q9BR82_HUMAN	PLAA	
STX5	RNF138		Q9BR82_HUMAN	PLAG1	
SUCLG1	RNF138		Q9BVM4_HUMAN	PLAGL1	
SUPT3H	RNF139		Q9BW98_HUMAN	PLAGL2	
SUSD2	RNF150		Q9BWW1_HUMAN	PLCB1	
SUSD3	RNF165		Q9BYA6_HUMAN	PLCD1	
SUSD3	RNF165		Q9BZV8_HUMAN	PLEKHA1	
SUSD4	RNF2		Q9NWC8_HUMAN	PLEKHA3	
SUV420H2	RNF34		Q9P181_HUMAN	PLEKHA7	
SVEP1	RNF4		Q9P1F0_HUMAN	PLEKHC1	
SYCE1	RNF4		Q9P1G6_HUMAN	PLEKHH1	
SYCE1	RNF41		Q9UI51_HUMAN	PLOD2	
SYCE1	RNF43		Q9UN38_HUMAN	PLP1	
SYCE2	RNF44		Q9UN38_HUMAN	PLP2	
TACR1	RNF6		R3HDML	PLXNA2	
TAF12	RNPS1		R3HDML	PLXNC1	
TAF13	ROBO1		RAB13	PMP2	
TAF15	ROD1		RAB21	PNPLA6	
TAF7L	RP11-145H9.1		RAB3C	PNRC1	
TAGAP	RP11-145H9.1		RAB3IP	POF1B	
TAGLN2	RP11-35N6.1		RAB40A	POLR3F	
TAGLN2	RPESP		RAB40A	POM121	
TAGLN2	RPS6KA3		RAB40A	POU3F2	
TALDO1	RPS6KA6		RAB40A	POU4F1	
TAOK2	RPS6KB1		RABGAP1L	PPAP2A	
TARBP1	RSBN1		RABGAP1L	PPAPDC2	
TARBP2	RSBN1L		RABGAP1L	PPARA	
TBC1D10A	RSBN1L		RAD54L	PPARGC1A	
TBC1D10A	RSPO3		RAD54L	PPARGC1B	
TBC1D17	RTF1		RAD54L	PPFIA3	
TBCD	RTF1		RAD9B	PPM1D	
TBN	RTN1		RAD9B	PPM1E	
TBP	RUNX1T1		RALGPS1	PPP1R11	
TBX18	RUNX1T1		RAP1A	PPP1R12A	
TCAP	RUNX2		RAPH1	PPP1R12B	
TCBA1	RWDD4A		RARRES1	PPP1R13B	
TCEAL2	RYBP		RASAL2	PPP1R15B	
TCF2	RYK		RASAL2	PPP1R3C	
TCF7	SACS		RASGRP2	PPP2CB	
TDRD10	SACS		RASGRP4	PPP2R1A	
TESC	SALL1		RASIP1	PPP2R5C	
TEX11	SALL1		RASIP1	PPP2R5E	
TEX11	SALL3		RASIP1	PPP3CB	
TEX13B	SAP130		RASIP1	PPP3CB	
TEX264	SAPS3		RASIP1	PPP3R1	
TFAP2A	SAR1B		RASL2_HUMAN	PPP6C	
TGFBRAP1	SATB2		RBBP8	PPRC1	

Plasma Microvesicles Predicted Targets			PBMC Predicted Targets		
Sanger miRBase	TargetScan	Common	Sanger miRBase	TargetScan	Common
TGIF1	SBK1		RBBP8	PPTC7	
THAP11	SCML2		RBBP8	PRC1	
THOC3	SCN1A		RBKS	PRDM1	
THRAP5	SCN3A		RBKS	PRDM4	
TIAM1	SCN3A		RBM3	PREI3	
TIMELESS	SCN3B		RBM32A	PRICKLE2	
TINAGL1	SCN4B		RBM38	PRKAA1	
TINAGL1	SCN8A		RBM6	PRKACA	
TLCD1	SCOC		RBMY1J	PRKACB	
TLCD1	SEC23A		RBMY1J	PRKAG2	
TLE1	SEC23IP		RBP4	PRKAR1A	
TLN1	SEC24A		RBP4	PRKAR2A	
TLR3	SELI		RBX1	PRKCA	
TLR6	SEMA3A		REPS1	PRKCD	
TM9SF2	SEMA3D		REPS2	PRKG1	
TM9SF2	SEMA4A		RFX4	PRM1	
TMCO5	SEMA4G		RFXANK	ProSAPiP1	
TMEM11	SEMA6D		RGS22	PRPF38A	
TMEM145	SEMA6D		RHCE	PRPF4B	
TMEM163	SEMA6D		RHCE	PRPS1	
TMEM16G	SENP5		RHCE	PRRT2	
TMEM16J	Septin2		RHCE	PRRT3	
TMEM185B	Septin6		RIBC1	PRX	
TMEM22	SERBP1		RIBC1	PSAP	
TMEM59	SERBP1		RILP	PSD	
TMEM69	SERP1		RILP	PSD3	
TMEM69	SERTAD1		RIMS1	PSKH1	
TMEM70	SESN1		RIN1	PSMD3	
TMEM85	SESN1		RIPK3	PSME3	
TMEM89	SET		RNF17	PTBP2	
TMEM93	SETD3		RNF190	PTCH1	
TMIGD2	SETD8		RNF32	PTEN	
TMSL8	SFRS11		RPA2	PTER	
TNFAIP8	SFRS12		RPA4	PTGER4	
TNFRSF10A	SFRS16		RPL21	PTGFRN	
TNFRSF18	SFRS6		RPL22L1	PTGS2	
TNFRSF19	SFXN5		RPL22L1	PTH	
TNFRSF6B	SGK		RPL22L1	PTK2B	
TNMD	SGMS1		RPL22L1	PTP4A1	
TNNC1	SGMS2		RPL23A	PTPN13	
TOX2	SH2B3		RPL27A	PTPN21	
TOX2	SH3BGRL2		RPL34	PTPN3	
TOX2	SH3BP4		RPL34	PTPRA	
TPM3	SH3D19		RPS12	PTPRD	
TPM3	SH3GL2		RPS15	PTPRE	
TPM3	SH3GL2		RPS4X	PTPRF	
TPT1	SH3PX3		RPS6KA1	PTPRG	
TRAF3IP3	SH3PXD2A		RPS6KA1	PURA	
TRAF3IP3	SH3PXD2A		RPS6KA6	PURB	
TRAF6	SH3RF1		RRAGA	PVRL1	
TRAF6	SHANK2		RRAGA	PXDN	
TRAV13-2	SHROOM2		RTN2	QKI	
TRI40_HUMAN	SIAH1		RTN2	R3HDM2	
TRIB3	SIDT1		RTP4	RAB10	
TRIB3	SIDT2		RUFY3	RAB11A	

Plasma Microvesicles Predicted Targets			PBMC Predicted Targets		
Sanger miRBase	TargetScan	Common	Sanger miRBase	TargetScan	Common
TRIM15	SIPA1L2		RXRG	RAB11FIP2	
TRIM15	SLC12A2		S100A10	RAB14	
TRIM29	SLC12A2		S100A10	RAB18	
TRIM3	SLC12A6		S100A13	RAB1A	
TRIM62	SLC13A3		S100A13	RAB21	
TRIM71	SLC16A6		S100B	RAB2B	
TRIM73	SLC19A2		S100P	RAB33B	
TRIM74	SLC1A1		SAA3P	RAB34	
TRIM9	SLC20A2		SALL4	RAB4B	
TSEN54	SLC23A2		SAMD11	RAB5B	
TSGA2	SLC24A4		SBSN	RAB6IP1	
TSGA2	SLC25A14		SCAMP3	RAB8B	
TSPAN14	SLC25A16		SCAMP3	RAB9A	
TSPAN14	SLC25A20		SCAND1	RAD23B	
TSPAN14	SLC25A35		SCAP	RAD9A	
TSPAN15	SLC25A37		SCN2A	RAF1	
TSPAN15	SLC25A37		SCN3A	RAI2	
TSPAN17	SLC26A4		SCN8A	RALGDS	
TSPYL1	SLC26A7		SCOC	RALGPS1	
TSPYL2	SLC2A14		SDCCAG8	RANBP10	
TTC1	SLC2A3		SDF2	RANBP3	
TTC7A	SLC36A1		SDS	RANBP9	
TTC8	SLC37A3		SEC11A	RAP1A	
TTC8	SLC38A2		SEC61A2	RAP1B	
TTC8	SLC39A1		SELENBP1	RAP2A	
TUBA1A	SLC39A10		SELENBP1	RAP2C	
TXNRD3	SLC39A8		SELK_HUMAN	RAPGEF4	
TYR	SLC4A4		SELO_HUMAN	RAPGEFL1	
U773_HUMAN	SLC4A4		SELV_HUMAN	RAPH1	
UBE1DC1	SLC4A4		SEMA4C	RARB	
UBE2M	SLC4A7		SEMA6D	RASA1	
UBQLNL	SLC5A3		SENP5	RASGEF1A	
UBXD1	SLC6A1		SENP8	RASGEF1B	
UFM1	SLC6A4		SERHL	RASL12	
UFM1	SLC6A6		SERPINA3	RASSF5	
UGT2B15	SLC7A11		SERPINB4	RBBP6	
UMOD	SLC7A2		SERPINI1	RBM16	
UMODL1	SLC8A1		SESN3	RBM24	
UQCRFS1	SLC9A2		SESN3	RBM35B	
URP2_HUMAN	SLC9A6		SETDB1	RBM6	
URP2_HUMAN	SLITRK1		SETX	RBM9	
USE1_HUMAN	SLITRK5		SGCA	RBMS1	
USP15	SMAD1		SGMS2	RBMS3	
USP16	SMAD3		SH2D6	RBPJ	
USP19	SMAD4		SH3GLB2	RBPMS2	
USP19	SMAD4		SH3GLB2	RCBTB1	
UTP14A	SMAD5		SHARPIN	RCN1	
UTP14A	SMAD7		SHARPIN	RCN2	
VAMP5	SMARCD1		SHARPIN	RCOR1	
VAMP8	SMARCD2		SHB	RECK	
VIT	SMURF1		SIAH1	REEP3	
VPS72	SMURF2		SIAH1	REEP4	
VPS72	SNCB		SIT1	RELN	
VWF	SNN		SIT1	REPS2	
VWF	SNN		SIX1	RET	

Plasma Microvesicles Predicted Targets			PBMC Predicted Targets		
Sanger miRBase	TargetScan	Common	Sanger miRBase	TargetScan	Common
WAPAL	SNN		SLC10A4	RFX1	
WAS	SNRK		SLC12A5	RFX4	
WDFY4	SNTB2		SLC17A2	RFXDC2	
WDR1	SNX13		SLC22A15	RGL1	
WDR18	SNX16		SLC22A20	RGS4	
WDR4	SNX22		SLC22A9	RHEBL1	
WDR4	SOBP		SLC25A11	RHOB	
WDR45	SOCS5		SLC25A35	RHOQ	
WDR45	SOCS6		SLC25A39	RICTOR	
WDR45	SOCS6		SLC25A39	RIMBP2	
WDR45	SORBS2		SLC26A10	RIMS1	
WDR62	SORT1		SLC26A10	RIN2	
WDR63	SOSTDC1		SLC26A6	RKHD2	
WDR63	SOX1		SLC26A6	RKHD3	
WDR63	SOX11		SLC26A6	RLF	
WDR64	SOX4		SLC27A2	RNASEH2C	
WDR67	SP1		SLC27A2	RND3	
WDR73	SP3		SLC2A1	RNF11	
WFDC5	SP6		SLC2A13	RNF111	
WFDC5	SP8		SLC2A6	RNF125	
WIPF1	SPAG7		SLC30A7	RNF128	
WNT11	SPEN		SLC35A2	RNF138	
WNT3	SPRED1		SLC39A5	RNF139	
WNT7A	SPRED1		SLC43A1	RNF165	
WWC1	SPRED1		SLC45A2	RNF167	
WWTR1	SPRYD3		SLC5A2	RNF170	
XR_015218.1	SPSB4		SLC6A18	RNF2	
XR_015754.1	SPTBN2		SLC6A18	RNF34	
XR_017939.1	SPTLC1		SLC7A10	RNF38	
XRCC1	SPTLC2		SLCO4A1	RNF4	
XRCC6	SRCAP		SLCO4A1	RNF41	
YAP1	SRGAP1		SLCO4A1	RNF43	
YIPF6	SRGAP3		SLFNL1	RNF44	
YIPF6	SRP19		SLURP1	RNF6	
ZAP70	SRPK1		SMARCA4	ROBO1	
ZAP70	SRPK1		SMC3	ROBO2	
ZAP70	SRPR		SMC3	ROCK1	
ZBTB45	SSFA2		SMCP	RP11-130N24.1	
ZCCHC13	SSH2		SMF_HUMAN	RP11-145H9.1	
ZCCHC13	SSX2IP		SMYD2	RP11-35N6.1	
ZDHHC1	ST8SIA4		SMYD2	RP13-102H20.1	
ZDHHC15	STAC2		SMYD2	RP5-1022P6.2	
ZDHHC16	STC1		SMYD2	RPESP	
ZDHHC16	STC2		SMYD3	RPS6KA2	
ZDHHC16	STIM1		SNAPC3	RPS6KA3	
ZDHHC16	STK19		SNAPC3	RPS6KA6	
ZDHHC18	STK33		SNCA	RPS6KB1	
ZDHHC19	STK39		SNCG	RS1	
ZDHHC19	STK39		SNCG	RSBN1	
ZDHHC2	STOX2		SNHG5	RSPO3	
ZDHHC6	STRBP		SNHG5	RTF1	
ZDHHC6	STRBP		SNHG5	RTN1	
ZIC5	STX1A		SNIP_HUMAN	RUNX1T1	
ZMYND8	STX5		SNRPA1	RUNX2	
ZNF202	STXBP1		SNRPC	RUNX3	

Plasma Microvesicles Predicted Targets			PBMC Predicted Targets		
**Sanger miRBase**	**TargetScan**	**Common**	**Sanger miRBase**	**TargetScan**	**Common**
ZNF219	STXBP3		SNRPC	RWDD4A	
ZNF259	STYX		SNTB2	RXRA	
ZNF275	STYX		SNX15	RYBP	
ZNF275	SULF1		SNX7	RYK	
ZNF277P	SUMO3		SNX7	SACS	
ZNF289	SUV420H2		SOCS1	SALL1	
ZNF32	SUV420H2		SON	SALL3	
ZNF32	SYBL1		SON	SAMD8	
ZNF365	SYNE1		SPACA4	SAPS1	
ZNF410	SYNJ1		SPACA4	SAPS2	
ZNF484	SYNPR		SPAG11A	SAPS3	
ZNF517	SYP		SPATA20	SAR1B	
ZNF524	SYPL1		SPATA5	SATB1	
ZNF524	SYT1		SPCS1	SATB2	
ZNF547	SYT10		SPCS1	SBF2	
ZNF553	TACC1		SPHK2	SCARA3	
ZNF645	TAF12		SPINK7	SCN1A	
ZNF688	TAF15		SPNS3	SCN1B	
ZNF74	TAF1L		SPSB3	SCN3A	
ZNF76	TAF5		SPTBN1	SCN3B	
ZNF76	TAF5		SPTBN1	SCN4B	
ZNF81	TAF5		SPTBN1	SCN8A	
ZNHIT3	TAF5		SRGAP2P1	SCOC	
ZPBP2	TAOK1		SRP19	SDC1	
ZRSR1	TBC1D10A		SRPK3	SEC22B	
ZSCAN2	TBC1D22B		SRPK3	SEC23IP	
ZW10	TBC1D24		SRPK3	SEC24A	
ZYX	TBC1D24		SRPK3	SELI	
	TBC1D4		SRPK3	SELT	
	TBL1XR1		SRPK3	SEMA3A	
	TBP		SRPK3	SEMA3D	
	TBPL1		SRPK3	SEMA3G	
	TCERG1		SRPK3	SEMA4C	
	TCERG1		SRPRB	SEMA4G	
	TCF12		SRPRB	SEMA6A	
	TCF7		SRPX	SEMA6B	
	TDRD7		SRPX	SEMA6D	
	TEX261		SRPX2	SENP5	
	TFAP2A		SSRP1	SEPHS2	
	TFAP2A		SSU72	Septin2	
	TFAP2D		SSX6	Septin6	
	TGFBR3		SSX6	Septin7	
	TGIF2		ST6GALNAC6	SERBP1	
	THRAP1		ST6GALNAC6	SERP1	
	THRAP2		ST6GALNAC6	SERTAD1	
	THRB		ST8SIA1	SESN1	
	TIMP3		STAMBPL1	SET	
	TIPARP		STARD10	SETD3	
	TJP1		STARD10	SETD5	
	TLE4		STARD3NL	SETD8	
	TLK1		STK19	SFMBT1	
	TLK2		STK19	SFRS11	
	TLL1		STK33	SFRS12	
	TLN2		STMN1	SFRS16	
	TLOC1		STOX2	SFRS2	

Plasma Microvesicles Predicted Targets			PBMC Predicted Targets		
Sanger miRBase	TargetScan	Common	Sanger miRBase	TargetScan	Common
	TLX3		STRBP	SFRS6	
	TMCC1		STRBP	SGCD	
	TMCC1		STRBP	SGEF	
	TMCC1		STRBP	SGK	
	TMEM135		STRBP	SGMS1	
	TMEM135		STX19	SGMS2	
	TMEM161B		STX5	SH2B3	
	TMEM165		STX5	SH3BGRL2	
	TMEM168		SUCLG1	SH3D19	
	TMEM16C		SUNC1	SH3GL2	
	TMEM2		SUNC1	SH3PX3	
	TMEM20		SUPT3H	SH3PXD2A	
	TMEM33		SURF5	SH3RF1	
	TMEM33		SUSD2	SHANK2	
	TMEM49		SUSD3	SIAH1	
	TMEM55A		SUSD3	SIDT1	
	TMEM64		SVEP1	SIDT2	
	TMEM64		SYCE1	SIPA1L2	
	TMEM68		SYCE1	SIX4	
	TMEM87B		SYCE2	SLAIN1	
	TMEM9		SYT6	SLC12A2	
	TMEM93		TACC2	SLC13A3	
	TMOD2		TACR1	SLC16A6	
	TMTC3		TACR2	SLC17A6	
	TNFRSF19		TAF12	SLC1A1	
	TNKS1BP1		TAF13	SLC1A2	
	TNKS2		TAF15	SLC20A2	
	TNPO1		TAGAP	SLC22A15	
	TNRC6A		TAGLN2	SLC23A2	
	TNRC6A		TAGLN2	SLC24A3	
	TNRC6B		TAGLN2	SLC24A4	
	TNRC6B		TALDO1	SLC25A14	
	TOB1		TAOK2	SLC25A16	
	TOP1		TAOK2	SLC25A20	
	TOP1		TARBP1	SLC25A35	
	TOX		TARBP2	SLC25A37	
	TP53INP1		TARBP2	SLC26A4	
	TP53INP1		TBC1D10A	SLC26A7	
	TP53INP1		TBC1D10A	SLC2A14	
	TPD52		TBC1D10A	SLC2A3	
	TRAF6		TBC1D17	SLC31A2	
	TRAF7		TBC1D2	SLC35F1	
	TRAK1		TBCD	SLC36A1	
	TRAM1		TBN	SLC37A3	
	TRIB2		TBP	SLC38A2	
	TRIM33		TBX18	SLC38A3	
	TRIM33		TBX18	SLC39A1	
	TRIM33		TBX18	SLC39A10	
	TRIM36		TCBA1	SLC39A8	
	TRIM71		TCEAL2	SLC4A4	
	TRIM9		TCEAL2	SLC4A7	
	TRPC3		TDRD10	SLC5A3	
	TRPC4AP		TESC	SLC5A7	
	TRPM6		TEX11	SLC6A1	
	TRPS1		TEX11	SLC6A4	

Plasma Microvesicles Predicted Targets			PBMC Predicted Targets		
Sanger miRBase	TargetScan	Common	Sanger miRBase	TargetScan	Common
	TRPS1		TFB1M	SLC6A6	
	TRPS1		TFDP2	SLC6A8	
	TSC22D2		TFDP2	SLC7A11	
	TSGA14		TGFBRAP1	SLC7A2	
	TSPAN14		TGIF1	SLC8A1	
	TSPAN5		TGIF1	SLC9A1	
	TSPAN7		TGIF1	SLC9A2	
	TSPYL2		TGM5	SLC9A6	
	TTC13		THAP11	SLITRK1	
	TTL		THOC3	SLITRK3	
	TTMB		TIMELESS	SLITRK6	
	TUBA1A		TIMELESS	SLMAP	
	TWF1		TINAGL1	SMAD1	
	UBE2A		TINAGL1	SMAD3	
	UBE2D1		TLCD1	SMAD4	
	UBE2E2		TLCD1	SMAD5	
	UBE2G1		TLR2	SMAD7	
	UBE2J1		TLR3	SMAP1L	
	UBE2J1		TLR3	SMARCA2	
	UBE2Q1		TLR5	SMARCC2	
	UBE2R2		TM9SF2	SMARCD1	
	UBE2V1		TM9SF2	SMARCD2	
	UBE4B		TMCO5	SMG7	
	UBE4B		TMEM11	SMOC1	
	UBFD1		TMEM112B	SMOC2	
	UBQLN1		TMEM125	SMURF1	
	UBQLNL		TMEM146	SMURF2	
	UBTD2		TMEM163	SMYD5	
	UBXD3		TMEM16G	SNIP	
	ULK1		TMEM185B	SNIP1	
	ULK2		TMEM22	SNN	
	UNC13A		TMEM58	SNPH	
	UNC84B		TMEM59	SNRK	
	USP15		TMEM69	SNTB2	
	USP15		TMEM69	SNX13	
	USP25		TMEM70	SNX16	
	USP25		TMEM85	SNX17	
	USP3		TMEM87B	SNX22	
	USP3		TMEM87B	SNX27	
	USP31		TMEM89	SOBP	
	USP31		TMEM93	SOCS1	
	USP37		TMIGD2	SOCS3	
	USP42		TMIGD2	SOCS5	
	USP9X		TMSL8	SOCS6	
	USP9X		TNFAIP8	SORBS2	
	USP9X		TNFAIP8	SORT1	
	VAMP1		TNFRSF19	SOSTDC1	
	VAMP8		TNFRSF19	SOX11	
	VANGL2		TNFRSF6B	SOX17	
	VAPB		TNK1	SOX21	
	VASH1		TNMD	SOX4	
	VASN		TNNC1	SOX5	
	VDAC1		TNXB	SOX6	
	VEGFA		TNXB	SP3	
	VEZF1		TNXB	SP6	

Plasma Microvesicles Predicted Targets			PBMC Predicted Targets		
Sanger miRBase	TargetScan	Common	Sanger miRBase	TargetScan	Common
	VGLL4		TOX2	SP8	
	VLDLR		TOX2	SPAG7	
	VNN1		TOX2	SPATA2	
	VTCN1		TPM3	SPEN	
	VTI1A		TPM3	SPG20	
	VTI1A		TPM3	SPHK2	
	WAPAL		TPT1	SPIRE1	
	WAPAL		TRAF2	SPRED1	
	WBP11		TRAF2	SPRY3	
	WDR22		TRAF3IP3	SPRY4	
	WDR23		TRAF3IP3	SPRYD3	
	WDR32		TRAF6	SPSB4	
	WDR47		TRAF6	SPTBN2	
	WDR68		TRAF6	SPTLC1	
	WDR68		TRAV12-1	SPTY2D1	
	WDTC1		TRAV13-2	SR140	
	WEE1		TRERF1	SRCAP	
	WIBG		TREX2	SRF	
	WIPF2		TRIM29	SRGAP1	
	WIPI2		TRIM3	SRGAP3	
	WNK3		TRIM71	SRP19	
	WNT3A		TRIM73	SRPK1	
	WNT5A		TRIM74	SRPR	
	WNT7A		TRIOBP	SRR	
	WSB1		TRO	SSFA2	
	WTAP		TSEN54	SSH2	
	WWP1		TSGA2	SSX2IP	
	WWP1		TSGA2	ST3GAL5	
	XAB1		TSPAN15	ST5	
	XKRX		TSPAN15	ST8SIA3	
	XPO4		TSPYL1	ST8SIA4	
	XPO4		TSPYL1	STAC2	
	YBX2		TSPYL2	STAT5B	
	YOD1		TTC1	STC1	
	YOD1		TTC5	STIM1	
	YPEL1		TTYH2	STK19	
	YTHDC1		TXNRD3	STK33	
	YTHDF3		TXNRD3	STK35	
	YTHDF3		UBAP2L	STK38	
	YTHDF3		UBASH3A	STK39	
	YWHAG		UBE1DC1	STMN1	
	YWHAH		UBE2M	STMN4	
	YWHAQ		UBE2M	STOX2	
	ZADH2		UBXD1	STRBP	
	ZBTB10		UCN	STX1A	
	ZBTB10		UCN	STX3	
	ZBTB38		UFD1L	STX5	
	ZBTB39		UFM1	STXBP1	
	ZBTB46		UHRF2	STXBP3	
	ZBTB9		ULK2	STYX	
	ZC3H6		UMOD	SULF1	
	ZCCHC14		UMODL1	SUMO1	
	ZCCHC14		UPK3A	SUMO3	
	ZCCHC2		UQCRFS1	SUPT16H	
	ZCCHC2		UROS	SUV420H2	

Plasma Microvesicles Predicted Targets			PBMC Predicted Targets		
Sanger miRBase	TargetScan	Common	Sanger miRBase	TargetScan	Common
	ZCCHC3		USP15	SUZ12	
	ZCCHC5		USP16	SV2A	
	ZDHHC17		USP19	SYBL1	
	ZDHHC18		USP19	SYCP3	
	ZDHHC6		VIT	SYDE1	
	ZEB2		VPS72	SYN1	
	ZER1		VPS72	SYNE1	
	ZFHX4		VPS72	SYNJ1	
	ZFHX4		VTA1	SYNPR	
	ZFPM2		VWF	SYPL1	
	ZFX		VWF	SYT1	
	ZFX		WAPAL	SYT10	
	ZFYVE1		WAS	SYT11	
	ZHX1		WAS	TACC1	
	ZHX3		WDFY4	TADA1L	
	ZIC1		WDR62	TAF12	
	ZIC5		WDR63	TAF15	
	ZIC5		WDR63	TAF1L	
	ZKSCAN1		WDR63	TAF4	
	ZMIZ1		WDR64	TAF5	
	ZMYM2		WDR73	TAF9B	
	ZMYM3		WFDC5	TAPT1	
	ZNF12		WFDC5	TBC1D10A	
	ZNF148		WNT11	TBC1D22B	
	ZNF148		WNT3	TBC1D24	
	ZNF148		WNT3	TBC1D4	
	ZNF148		WNT7A	TBK1	
	ZNF217		WRNIP1	TBL1XR1	
	ZNF236		WWC1	TBP	
	ZNF238		XG	TBPL1	
	ZNF238		XR_015218.1	TCERG1	
	ZNF289		XR_015754.1	TCF12	
	ZNF3		XR_017939.1	TDRD7	
	ZNF365		XR_018421.1	TESK2	
	ZNF367		XRCC6	TEX261	
	ZNF385		XYLT2	TFAP2A	
	ZNF410		YAP1	TFAP2C	
	ZNF423		YIPF6	TFAP2D	
	ZNF436		YIPF6	TFAP4	
	ZNF462		YPEL4	TGFBR2	
	ZNF512B		ZAP70	TGFBR3	
	ZNF532		ZBTB45	TGIF1	
	ZNF532		ZCCHC13	TGIF2	
	ZNF533		ZCCHC13	TGM3	
	ZNF608		ZCCHC7	TGOLN2	
	ZNF609		ZCWPW1	THAP3	
	ZNF618		ZDHHC15	THRAP1	
	ZNF622		ZDHHC16	THRAP2	
	ZNF629		ZDHHC16	THRB	
	ZNF650		ZDHHC16	TIA1	
	ZNF650		ZDHHC16	TJP1	
	ZNF654		ZDHHC16	TLE4	
	ZNF654		ZDHHC18	TLK1	
	ZNF664		ZDHHC19	TLK2	
	ZNF667		ZDHHC19	TLL1	

Plasma Microvesicles Predicted Targets			PBMC Predicted Targets		
Sanger miRBase	TargetScan	Common	Sanger miRBase	TargetScan	Common
	ZNF697		ZDHHC2	TLN2	
	ZNF697		ZDHHC2	TLX3	
	ZNF697		ZDHHC6	TM9SF3	
	ZNF704		ZDHHC6	TMCC1	
	ZSCAN21		ZIC5	TMEM135	
	ZSWIM3		ZMYM3	TMEM168	
	ZXDA		ZMYM3	TMEM16A	
	ZXDB		ZNF202	TMEM16C	
	ZXDC		ZNF259	TMEM2	
	ZXDC		ZNF275	TMEM20	
	ZYX		ZNF275	TMEM24	
			ZNF277P	TMEM32	
			ZNF32	TMEM33	
			ZNF32	TMEM47	
			ZNF335	TMEM49	
			ZNF410	TMEM55A	
			ZNF484	TMEM63B	
			ZNF517	TMEM64	
			ZNF524	TMEM68	
			ZNF547	TMEM87A	
			ZNF582	TMEM87B	
			ZNF593	TMEM93	
			ZNF614	TMEPAI	
			ZNF645	TMF1	
			ZNF688	TMOD2	
			ZNF711	TMPRSS11F	
			ZNF81	TMTC3	
			ZNHIT3	TNFRSF12A	
			ZRSR1	TNIP1	
			ZSCAN2	TNKS	
			ZW10	TNKS1BP1	
			ZW10	TNKS2	
			ZWINT	TNPO1	
			ZYX	TNPO2	
				TNRC6A	
				TNRC6B	
				TOB1	
				TOM1L2	
				TOP1	
				TOR1B	
				TP53INP1	
				TP53INP2	
				TP73L	
				TPD52	
				TRAF6	
				TRAK1	
				TRAK2	
				TRAM1	
				TRIB2	
				TRIM2	
				TRIM33	
				TRIM36	
				TRIP10	
				TRPC3	
				TRPS1	

PBMC Predicted Targets		
Sanger miRBase	TargetScan	Common
	TSC1	
	TSC22D2	
	TSC22D3	
	TSGA14	
	TSHZ3	
	TSPAN5	
	TSPAN7	
	TSPYL2	
	TTC13	
	TTL	
	TTMB	
	TTYH3	
	TUBA1A	
	TUT1	
	TWF1	
	TXNDC12	
	TXNDC4	
	UBAP1	
	UBE2A	
	UBE2D1	
	UBE2D2	
	UBE2E2	
	UBE2E3	
	UBE2G1	
	UBE2J1	
	UBE2Q1	
	UBE2R2	
	UBE2V1	
	UBE2W	
	UBE2Z	
	UBE4B	
	UBE4B	
	UBFD1	
	UBL3	
	UBQLN2	
	UBQLNL	
	UBTD2	
	UBTF	
	UBXD3	
	UCP3	
	UHMK1	
	ULK1	
	ULK2	
	UNC13A	
	USP14	
	USP15	
	USP2	
	USP25	
	USP3	
	USP31	
	USP32	
	USP33	
	USP37	
	USP42	
	USP48	

PBMC Predicted Targets		
Sanger miRBase	TargetScan	Common
	USP6	
	USP9X	
	UST	
	UTX	
	UTY	
	VAMP1	
	VAMP2	
	VAMP8	
	VANGL2	
	VASN	
	VAT1	
	VAV2	
	VCPIP1	
	VDAC1	
	VEGFA	
	VEZF1	
	VGLL4	
	VLDLR	
	VNN1	
	VPS37A	
	VPS37B	
	VPS37C	
	VPS4B	
	VTCN1	
	VTI1A	
	WAPAL	
	WASF2	
	WBP11	
	WBP2	
	WDFY3	
	WDR1	
	WDR20	
	WDR22	
	WDR26	
	WDR32	
	WDR44	
	WDR45L	
	WDR47	
	WDR68	
	WDTC1	
	WEE1	
	WHSC1	
	WHSC1L1	
	WIBG	
	WIPF2	
	WIPI2	
	WISP1	
	WNK3	
	WNT1	
	WNT2B	
	WNT3	
	WNT3A	
	WNT5A	
	WNT7A	
	WSB1	

PBMC Predicted Targets		
Sanger miRBase	TargetScan	Common
	WWP1	
	XAB1	
	XKRX	
	XPO4	
	YES1	
	YOD1	
	YPEL1	
	YTHDC1	
	YTHDF2	
	YTHDF3	
	YWHAE	
	YWHAH	
	YWHAQ	
	ZAK	
	ZBTB10	
	ZBTB38	
	ZBTB39	
	ZBTB4	
	ZBTB46	
	ZBTB9	
	ZC3H12B	
	ZC3H6	
	ZC3H7B	
	ZCCHC14	
	ZCCHC2	
	ZCCHC3	
	ZCCHC5	
	ZDHHC17	
	ZDHHC18	
	ZDHHC6	
	ZDHHC7	
	ZDHHC9	
	ZEB1	
	ZEB2	
	ZER1	
	ZFAND5	
	ZFHX4	
	ZFP91	
	ZFPM2	
	ZFX	
	ZFYVE1	
	ZFYVE21	
	ZFYVE26	
	ZFYVE9	
	ZHX1	
	ZHX2	
	ZHX3	
	ZIC1	
	ZIC5	
	ZKSCAN1	
	ZKSCAN2	
	ZMIZ1	
	ZMYM2	
	ZMYND11	
	ZNF12	

PBMC Predicted Targets		
Sanger miRBase	TargetScan	Common
	ZNF148	
	ZNF189	
	ZNF217	
	ZNF236	
	ZNF238	
	ZNF289	
	ZNF3	
	ZNF365	
	ZNF367	
	ZNF403	
	ZNF410	
	ZNF423	
	ZNF436	
	ZNF462	
	ZNF474	
	ZNF512B	
	ZNF518	
	ZNF532	
	ZNF533	
	ZNF608	
	ZNF609	
	ZNF622	
	ZNF644	
	ZNF650	
	ZNF654	
	ZNF664	
	ZNF667	
	ZNF697	
	ZNF704	
	ZNF711	
	ZNF784	
	ZNF800	
	ZRANB2	
	ZSWIM3	
	ZXDC	
	ZYG11B	
	ZYX	
