# Supplementary material for: Detection of microRNA Expression in Human Peripheral Blood Microvesicles
Source: PLoS One. 2008 Nov 11;3(11):e3694. doi: 10.1371/journal.pone.0003694 (PMC2577891; doi:10.1371/journal.pone.0003694)
Supplement: Table S3 — Comparison of miRNA expression between plasma microvesicles and PBMC. In order to test the differences of miRNA expression between plasma microvesicles and PBMC, data were analyzed using linear mixed models. The p-values were generated from the model based on the estimated difference and sample variation. A bonferroni adjustment for multiple comparisons (72 comparisons) to control type I error to reduce the number of false positives was performed, therefore “p-value” was considered as significant if p-value<0.05/72 = 0.0006. Fold-change was calculated based on the estimated mean difference (2∧(−ΔCT)). (0.01 MB PDF) [file pone.0003694.s003.pdf]

Detector_Name	Plasma Microvesicles Mean CT	PBMC Mean CT	delta CT (Plasma MV- PBMC)	Plasma Microvesicle Fold Change	PBMC Fold Change	p-Value
hsa-let-7a	34.1657	34.6615	-0.4145	1.3328	0.7503	0.031855987
hsa-let-7b	32.3929	33.2312	-0.757	1.6900	0.5917	4.25E-06
hsa-let-7g	33.6069	31.9889	1.6993	0.3079	3.2474	4.99E-09
hsa-miR-015a	36.0264	34.4178	1.6899	0.3099	3.2263	3.25E-07
hsa-miR-015b	31.4483	32.0744	-0.5448	1.4588	0.6855	0.000371404
hsa-miR-016	27.7458	26.5689	1.2581	0.4181	2.3918	1.35E-10
hsa-miR-017-5p	31.3119	32.4196	-1.0264	2.0369	0.4909	1.26E-07
hsa-miR-019a	32.1131	29.9671	2.2273	0.2136	4.6826	2.86E-12
hsa-miR-019b	29.4539	27.8512	1.6841	0.3112	3.2134	7.12E-12
hsa-miR-020a	29.1097	28.3667	0.8243	0.5648	1.7707	3.74E-06
hsa-miR-020b	34.9782	33.8442	1.2152	0.4307	2.3217	0.000852341
hsa-miR-021	34.3397	32.3467	2.0743	0.2375	4.2114	4.23E-08
hsa-miR-024	27.7870	28.3626	-0.4943	1.4086	0.7099	0.000137267
hsa-miR-025	36.2723	35.8526	0.501	0.7066	1.4152	0.069149461
hsa-miR-026a	27.6115	27.3268	0.366	0.7759	1.2888	0.003310506
hsa-miR-026b	32.2939	31.1691	1.206	0.4335	2.3070	5.44E-06
hsa-miR-027a	33.3390	32.9954	0.4249	0.7449	1.3425	0.101287776
hsa-miR-027b	34.9732	37.1277	-2.0732	4.2082	0.2376	1.20999E-05
hsa-miR-029a	37.1277	31.8757	5.3333	0.0248	40.3165	9.70E-28
hsa-miR-030a-5p	32.8490	32.1146	0.8157	0.5681	1.7602	4.98E-07
hsa-miR-030b	30.5581	30.7201	-0.0807	1.0575	0.9456	0.389875101
hsa-miR-030c	30.9372	31.0159	0.002625	0.9982	1.0018	0.97816881
hsa-miR-030d	33.8832	32.9862	0.9782	0.5076	1.9700	0.000154741
hsa-miR-032	29.5442	31.8053	-2.1798	4.5309	0.2207	8.52E-09
hsa-miR-092	29.7522	30.5540	-0.7205	1.6478	0.6069	1.17E-09
hsa-miR-093	29.9299	30.3308	-0.3196	1.2480	0.8013	0.043597802
hsa-miR-096	31.4698	32.7342	-1.1831	2.2706	0.4404	0.000386324
hsa-miR-103	33.4330	34.6453	-1.131	2.1901	0.4566	1.99E-08
hsa-miR-106a	32.2734	33.3104	-0.9557	1.9395	0.5156	4.92E-07
hsa-miR-106b	33.7428	32.5076	1.3165	0.4015	2.4906	1.28E-07
hsa-miR-125a	30.9410	31.6341	-0.6118	1.5282	0.6544	0.02785181
hsa-miR-126	28.0763	29.1003	-0.9427	1.9221	0.5203	2.61E-11
hsa-miR-126*	31.1668	32.1346	-0.8865	1.8487	0.5409	0.001314585
hsa-miR-133b	35.0417	36.8666	-1.7435	3.3485	0.2986	4.05E-06
hsa-miR-140	30.8069	28.8112	2.077	0.2370	4.2193	2.87E-17
hsa-miR-142-3p	33.4503	28.9770	4.5545	0.0426	23.4986	1.91E-22
hsa-miR-142-5p	33.8091	32.4730	1.4174	0.3744	2.6710	9.83E-08
hsa-miR-146a	27.0517	26.9589	0.1741	0.8863	1.1283	0.241458101
hsa-miR-146b	30.8772	26.4438	4.5147	0.0437	22.8592	1.81E-22
hsa-miR-150	30.4759	24.6519	5.9053	0.0167	59.9339	7.01E-21
hsa-miR-151	30.4093	32.2909	-1.8003	3.4829	0.2871	2.24E-21
hsa-miR-155	32.7730	29.6445	3.2098	0.1081	9.2522	6.61E-23
hsa-miR-181d	36.9125	35.1012	1.8926	0.2693	3.7130	5.30E-07
hsa-miR-182*	34.5125	35.1958	-0.6021	1.5179	0.6588	0.04206113
hsa-miR-183	32.4542	35.1156	-2.5801	5.9798	0.1672	7.17E-08
hsa-miR-186	31.3118	31.4537	-0.06062	1.0429	0.9589	0.659198081
hsa-miR-191	26.6389	27.2756	-0.7068	1.6322	0.6127	0.00047708
hsa-miR-195	33.5406	32.4568	1.1652	0.4459	2.2426	0.000055077
hsa-miR-196b	33.8919	34.5622	-0.589	1.5042	0.6648	0.052904716
hsa-miR-197	30.2200	32.2212	-1.9199	3.7840	0.2643	5.34E-18
hsa-miR-199a*	33.5873	35.4571	-1.7886	3.4548	0.2895	3.21E-09
hsa-miR-222	27.6926	29.7721	-1.9982	3.9950	0.2503	1.18E-21
hsa-miR-223	21.6367	21.1618	0.5561	0.6801	1.4703	2.99379E-05
hsa-miR-302b	33.2311	34.3343	-1.0219	2.0306	0.4925	0.003731969
hsa-miR-320	32.3865	33.8670	-1.3991	2.6374	0.3792	1.63E-16
hsa-miR-328	30.1600	32.9643	-2.723	6.6024	0.1515	1.64E-21
hsa-miR-331	31.2031	31.2598	0.02458	0.9831	1.0172	0.843135202
hsa-miR-340	35.7147	35.5380	0.258	0.8362	1.1958	0.297093531
hsa-miR-342	30.0486	29.3479	0.782	0.5816	1.7195	3.06E-07
hsa-miR-345	36.5137	34.7192	1.8759	0.2725	3.6703	1.26E-08
hsa-miR-374	34.5105	32.6775	1.9143	0.2653	3.7693	8.01E-15
hsa-miR-376a	35.4948	36.2219	-0.6458	1.5646	0.6391	0.048003434
hsa-miR-423	33.9374	34.8228	-0.8041	1.7461	0.5727	0.001362239
hsa-miR-425-3p	34.7742	35.8236	-0.9681	1.9563	0.5112	0.00084906
hsa-miR-425-5p	33.2364	33.8810	-0.5633	1.4776	0.6768	0.00179719
hsa-miR-484	26.5575	28.1305	-1.4917	2.8122	0.3556	0.009046242
hsa-miR-486	28.7317	31.5663	-2.7533	6.7426	0.1483	4.31E-20
hsa-miR-532	36.3670	34.2064	2.2419	0.2114	4.7302	6.15E-10
hsa-miR-574	29.7788	31.9944	-2.1344	4.3905	0.2278	3.05E-06
hsa-miR-628	33.6589	34.2345	-0.4943	1.4086	0.7099	0.033363855
hsa-miR-643	35.6735	37.1666	-1.4118	2.6607	0.3758	0.00130314

Linear mixed models were used and p-values were generated from the model based on the estimated difference and sample variation. "p-value" was considered as significant if p-value<0.05/72=0.0006
